# MiR-92a/KLF4/p110δ regulates titanium particles-induced macrophages inflammation and osteolysis

**DOI:** 10.1038/s41420-022-00999-2

**Published:** 2022-04-13

**Authors:** Zhenkang Wen, Sipeng Lin, Changchuan Li, Zhuji Ouyang, Zhong Chen, Shixun Li, Yuxi Huang, Wenqiang Luo, Zhongcan Zheng, Peidong Guo, Manyuan Kuang, Yue Ding

**Affiliations:** Department of Orthopedic Surgery, Sun Yat-sen Memorial Hospital, Sun Yat-sen University, Guangzhou, China

**Keywords:** Chronic inflammation, Bone development

## Abstract

As total joint replacement is widely applied for severe arthropathy, peri-prosthetic aseptic loosening as one of the main causes of implant failure has drawn wide attention. Wear particles such as titanium particles (TiPs) derived from prosthesis can initiate macrophages inflammation and sequentially activate osteoclasts, which results in bone resorption and osteolysis for long-term. Therefore, inhibiting wear particles induced macrophages inflammation is considered as a promising therapy for AL. In this research, we found that the inhibition of p110δ, a member of class IA PI3Ks family, could significantly dampen the TiPs-induced secretion of TNFα and IL-6. By the transfection of siRNA targeting p110δ, we confirmed that p110δ was responsible for TNFα and IL-6 trafficking out of Golgi complex without affecting their expression in TiPs-treated macrophages. As the upstream transcription-repressor of p110δ, Krüppel-like factor 4 (KLF4), targeted by miR-92a, could also attenuate TiPs-induced inflammation by mediating NF-κB pathway and M1/M2 polarization. To further ascertain the roles of KLF4/p110δ, TiPs-induced mice cranial osteolysis model was established and vivo experiments validated that KLF4-knockdown could exacerbate TiPs-induced osteolysis, which was strikingly ameliorated by knockdown of p110δ. In summary, our study suggests the key role of miR-92a/KLF4/p110δ signal in TiPs-induced macrophages inflammation and osteolysis.

## Introduction

As an effective therapy to restore joint function, total joint replacement is an alternative for severe arthropathy and the demand for total hip or knee arthroplasty is growing rapidly [1]. However, the life span of prosthesis is limited with various reasons contributing to implants failure and among that, aseptic loosening (AL) is one of the main causes [2, 3]. Mechanically, dissociative wear debris like titanium particles (TiPs) derived from prosthesis can activate macrophages to produce inflammatory cytokines including tumor necrosis factor alpha (TNF-α) and interleukin-6 (IL-6), which further activates osteoclasts and leads to bone resorption [4, 5]. Therefore, alleviating the wear particles induced macrophages inflammation is considered as an underlying therapy for aseptic loosening [6].

Class I PI3Ks family, which can be further divided into class IA (p110α, p110β, p110δ) or class IB (p110γ), is ubiquitously reported to control cell survival, migration, differentiation and it involves in the progress of various diseases including skeletal-related inflammatory diseases [7–10]. Former studies demonstrate that p110α/β is positively relative to TiPs-induced TNFα production and the development of osteolysis [11, 12]. Abundant in leukocytes [13], p110δ, as another critical factor involving in inflammatory response and cell migration upon inflammation [14–16], is also reported to be responsible for TNFα trafficking and secretion by controlling the fission of Golgi complex upon pro-inflammatory stimulus [17, 18]. However, its concrete role in wear particles induced peri-prosthetic aseptic loosening is remained unknown.

Krüppel-like factor 4 (KLF4) containing three zinc-finger motifs is a member of SP/KLF factors, which functions as a transcription factor in diverse physiological process [19]. Apart from known as a tumor mediator [20], KLF4 can mediate inflammatory pathways and it involves in the development of diseases such as atherosclerosis, kidney injury, osteoarthritis [21–23]. As an underlying anti-inflammatory factor, it has been reported that KLF4 can attenuate macrophages inflammation by inactivating NF-κB pathway and manage macrophages polarization, cells apoptosis and other biological activities [24–27]. As small single-stranded and non-coding RNAs that are widely studied nowadays, microRNAs directly target the specific mRNAs to interrupt their expression. It is reported that KLF4 can be mediated by various microRNAs such as miR-148-3p, miR-152-3p and miR-92a to further participate in the downstream physiological process [28–30]. Nevertheless, the relation between KLF4 and p110δ is unexplored, and whether KLF4 can be targeted by a specific microRNA to further regulate the development of aseptic loosening is obscure.

In this study, we revealed the pro-inflammatory role of p110δ, which was mediated by miR-92a/KLF4 in TiPs-stimulated macrophages. Firstly, we screened p110δ as a TiPs-induced inflammation relative factor and confirmed that its expression was facilitated toward TiPs stimulation. With the application of IC87114 or si-p110δ, we found that p110δ involved in the transport of TNFα and IL-6 from Golgi complex to plasma membrane. KLF4, targeted by miR-92a, was predicted as a transcriptional repressor of p110δ and it could also attenuate the TiPs activated NF-κB pathway and M1/M2 macrophages polarization ratio. In vivo experiments, the role of KLF4 and p110δ were further confirmed in TiPs-induced mice cranial osteolysis, suggesting p110δ as a pro-osteolysis factor while KLF4 was an anti-osteolysis factor.

## Results

### P110δ might be a significant mediator in TiPs-induced macrophages inflammation

**Fig. 1:**
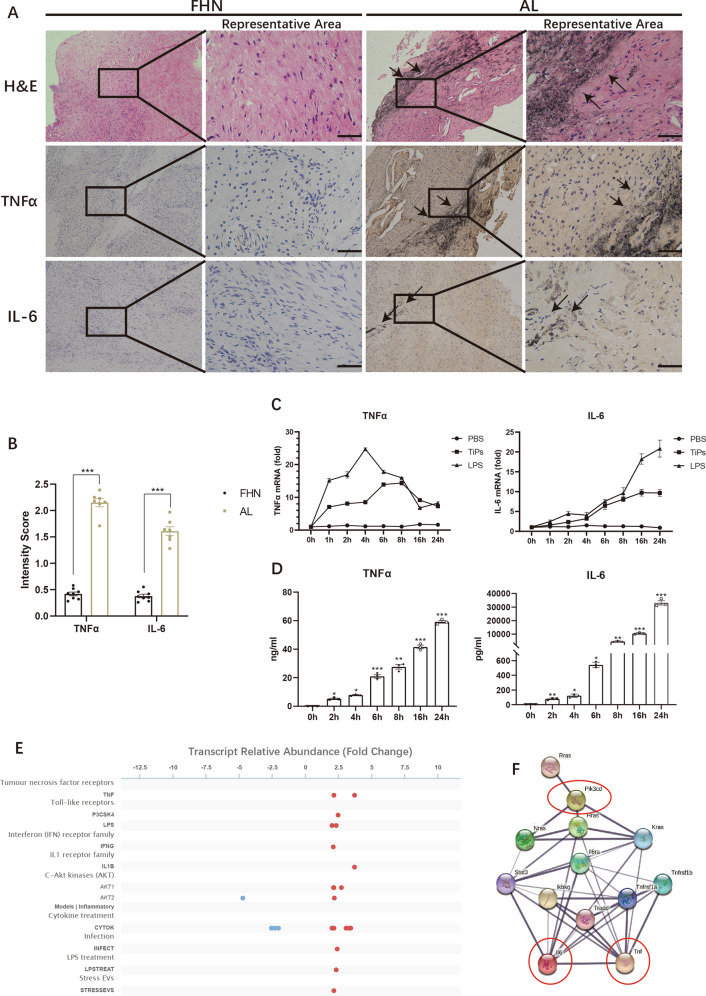
P110δ might be a significant mediator in TiPs-induced macrophages inflammation. **A** Representative image of Hematoxylin-Eosin (H&E) staining and immunohistochemical (IHC) staining for TNFα and IL-6 of human synovial membrane from femoral head necrosis (FHN) patients and aseptic loosening (AL) patients. The black bar, 50 μm. **B** Average intensity score (IS) of IHC staining was calculated by Bresalier’s analysis. **C**, **D** The mRNA expressions (**C**) and serum levels (**D**) of TNFα and IL-6 in RAW264.7 were ascertained by qPCR and ELISA assay upon different stimulus for various periods. **E** The Signaling Pathways Project (SPP) database indicated that the expression of p110δ was elevated upon various pro-inflammatory stimulation including TNFα, IL-1β, interferon γ, LPS, etc. **F** Protein–protein interaction network (PPIN) from STRING database depicted a strong connection between p110δ and pro-inflammatory cytokines. All data were concluded from at least three independent assays. Statistic data were displayed as mean ± SEM and were conducted unpaired *t* test analysis or one-way ANOVA analysis to determine significant difference. *p < 0.05, **p < 0.01, ***p < 0.001 compared with the negative group.

We firstly gathered synovial membranes from AL and FHN patients for Immunohistochemistry (IHC) staining to detected the productions of TNFα and IL-6. We found stronger TNFα and IL-6 staining with higher IS score (2.15 ± 0.08 vs 0.42 ± 0.04 for TNFα, *p* < 0.05; 1.61 ± 0.09 vs 0.38 ± 0.03 for IL-6, *p* < 0.05) in AL group upon TiPs infiltration (Fig. 1A, B). Since that TiPs could aggravate the inflammation in synovial membrane, we next conducted vitro assays and confirmed that TiPs stimulation led to elevated production of TNFα and IL-6 in macrophages (Fig. 1C, D). These data verified the upregulating levels of TNF-α and IL-6 in synovial membranes from aseptic loosening patients and TiPs-stimulated macrophages.

In our previous study, we have performed RNA-seq to profile the mRNA expression network of 4 h TiPs stimulated RAW264.7 [31]. A heat map with top 50 differential expression genes (DEGs) and KEGG pathway analysis for the top 20 pathways, including inflammation-relative pathways, were depicted (Fig. S1A, B). After screening the DEGs, we noticed that the expression of p110δ, with gene ID named Pik3cd, was nearly double upon TiPs stimulation. According to the Signaling Pathways Project database (SPP, https://www.signalingpathways.org/index.jsf) and STRING database (https://string-db.org/), we discovered that the production of p110δ was upregulated upon various inflammatory stimulation including TNFα, IL-1β, interferon γ, and LPS, and there was a strong connection between p110δ and pro-inflammatory factors. (Fig. 1E, F). These data implied that p110δ might also function as the key role in TiPs-induced macrophages inflammation.

### Inhibition of p110δ could reduce TNFα and IL-6 secretion in TiPs-stimulated macrophages

**Fig. 2:**
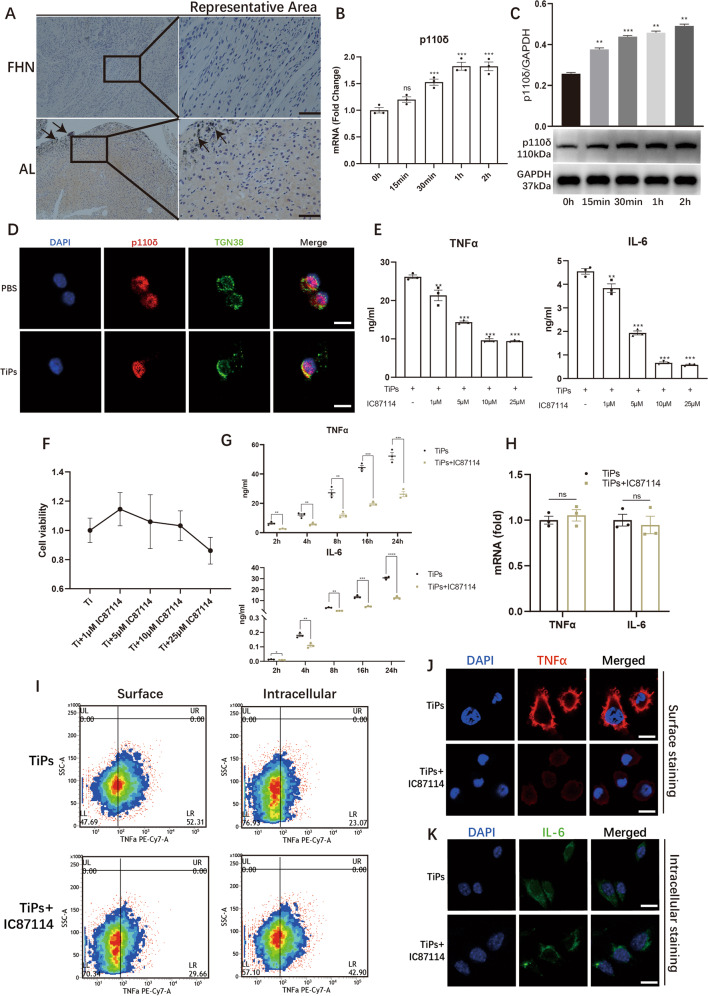
Inhibition of p110δ could reduce TNFα and IL-6 secretion in TiPs-stimulated macrophages. **A** IHC staining to detect p110δ expression of synovial membranes from FHN patients and AL patients. The black bar, 50 μm. **B**, **C** mRNA (**B**) and protein productions (**C**) of p110δ in 2 h TiPs-stimulated RAW264.7. **D** TiPs stimulation resulted in the co-localization of p110δ with TGN38. The white bar, 10 μm. **E** After 8 h TiPs and IC87114 (selective inhibitor of p110δ) treatment, serum levels of TNFα and IL-6 from RAW264.7 were detected by ELISA assay. **F** Cell viability upon 16 h TiPs treatment with or without IC87114. **G** TNFα and IL-6 secretions were detected after TiPs stimulation with or without 10 μM IC87114. **H** mRNA expressions of TNFα and IL-6 upon TiPs stimulation with or without 10 μM IC87114. **I** Surface TNFα and intracellular TNFα were analysed respectively by FCM assay after 2 h TiPs stimulation with or without 10 μM IC87114. For surface TNFα detection, permeabilization was not applied and 10 μM TAPI-1 (TACE inhibitor) was employed to block the cleavage of TNFα. The surface TNFα detection could reflect the secretion of TNFα. For intracellular detection, permeabilization was conducted without TAPI-1 treatment. And the intracellular detection could reflect the cellular residual TNFα. **J** Co-treated with 10 μM TAPI-1, surface TNFα staining was achieved by confocal microscopy to reflect the secretion of TNFα. The white bar, 10 μm. **K** Cellular IL-6 staining result was achieved by confocal microscopy to reflect the intracellular distribution of IL-6. The white bar, 10 μm. All data were concluded from at least three independent assays. Statistic data were displayed as mean ± SEM and were conducted unpaired *t* test analysis or one-way ANOVA analysis to determine significant difference. *p < 0.05, **p < 0.01, ***p < 0.001 compared with the negative group.

We then detected whether the expression of p110δ differed upon TiPs stimulation. IHC staining was performed, showing that synovial membranes from AL group achieved a stronger p110δ staining result, with a higher average IS than that of FHN group (1.46 ± 0.06 vs 0.42 ± 0.06, *p* < 0.05) (Figs. 2A and S1C). Next, RAW264.7 were treated with TiPs to explore the production of p110δ. As revealed in western blot (WB) and Qualitive real-time PCR (qPCR) assays, mRNA and protein levels of p110δ were rapidly augmented within 2 h TiPs stimulation, which implied that p110δ might be a pro-inflammatory factor upon TiPs stimulation (Fig. 2B, C). By confocal microscopy assay, the intracellular distribution of p110δ was observed and it indicated a co-localization between p110δ and Golgi complex upon 2 h TiPs stimulation (Fig. 2D).

Next, the activity of p110δ was inhibited by IC87114, a p110δ selective inhibitor, to explore the role of p110δ. Co-treated with TiPs for 8 h, IC87114 led to attenuated TNFα and IL-6 secretions and the concentration of 10 μM exhibited the most promising inhibitory effect without affecting cell viability (Fig. 2E–G). But when qPCR assay was conducted, IC87114 demonstrated no impact on the mRNA expressions of TNFα and IL-6 (Fig. 2H). These results implied that p110δ may affect the post-transcriptional modification process or trafficking process of TNFα and IL-6.

Since that TNFα needed to be cleaved by TNFα-converting enzyme (TACE) into mature form for secretion, we could detect the surface or intracellular distribution of TNFα by the application of TAPI-1, an inhibitor of TACE. Flow cytometry (FCM) assay revealed that TiPs-induced surface level of TNFα declined from 52.82 ± 2.28 to 27.57 ± 1.37 while intracellular TNFα was significantly cemented due to the presence of IC87114, indicating that the inhibition of p110δ increased residual TNFα and reduced the secreted TNFα (Figs. 2I and S1D). Correspondingly, Immunofluorescence (IF) staining was conducted and we found that inhibition of p110δ led to mild TNFα surface staining (Fig. 2J). Due to IL-6 was secreted directly as a soluble protein, the surface level of IL-6 was undetectable. Therefore, we directly measured the intracellular IL-6 distribution and found that in solely TiPs stimulated group, the distribution of IL-6 was diffused while IL-6 was restricted mainly around the nucleus in TiPs+IC87114 group (Fig. 2K). Considering the co-localization of p110δ and Golgi complex upon TiPs stimulation, we assumed that p110δ might be responsible for the transport of TNFα and IL-6 out of Golgi complex.

### Knockdown p110δ attenuated TNFα and IL-6 secretions by disturbing their trafficking out of Golgi complex in TiPs-stimulated macrophages

**Fig. 3:**
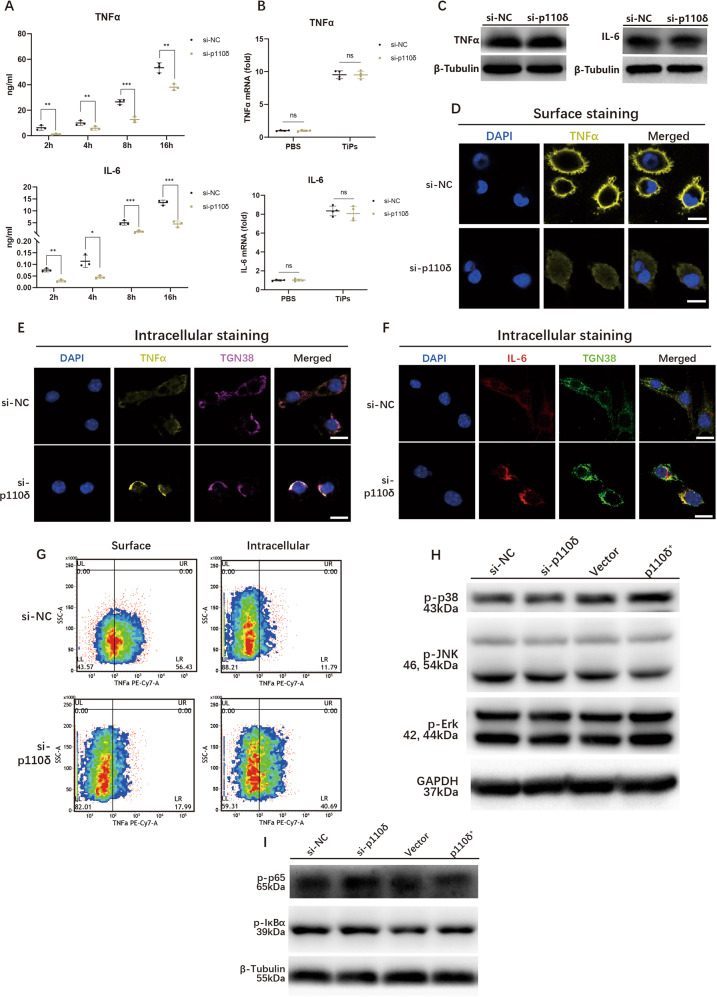
Knockdown p110δ attenuated TNFα and IL-6 secretions by disturbing their trafficking out of Golgi complex in TiPs-stimulated macrophages. **A** TiPs-induced secretions of TNFα and IL-6 from p110δ-knockdown macrophages were detected by ELISA assay. **B** mRNA productions of TNFα and IL-6 were examined by qPCR assay in p110δ-knockdown RAW264.7 upon 8 h TiPs stimulation. **C** Protein levels of TNFα and IL-6 were analysed by WB assay after 8 h TiPs stimulation with Brefeldin A (BFA), an inhibitor to hinder the transit from Endoplasmic Reticulum (ER) to Golgi complex. **D**, **E** After treated with TiPs for 2 h, p110δ-knockdown RAW264.7 were imaged with confocal microscopy to detect TNFα distribution. **D** Macrophages were co-treated with TiPs and 10 μM TAPI-1 to detect the surface distribution without permeabilization. It reflected the secretion of TNFα. The white bar, 10 μm. **E** Permeabilization was performed to detect the intracellular distribution of TNFα, which reflecting the cellular residual TNFα. The white bar, 10 μm. **F** After treated with TiPs for 2 h, p110δ-knockdown RAW264.7 were imaged with confocal microscopy to detect cellular IL-6 distribution. The white bar, 10 μm. **G** Surface TNFα and intracellular TNFα were analysed respectively by FCM assay after 2 h TiPs stimulation with or without 10 μM IC87114. For surface TNFα detection, permeabilization was not applied and 10 μM TAPI-1 (TACE inhibitor) was employed to block the cleavage of TNFα. The surface TNFα detection could reflect the secretion of TNFα. For intracellular detection, permeabilization was conducted without TAPI-1 treatment. And the intracellular detection could reflect the cellular residual TNFα. **H**, **I** Activation of MAPK signaling pathway (**H**) and NF-κB signaling pathway (**I**) were examined after 1 h TiPs stimulation in p110δ-knocdown or p110δ-overexpressed macrophages. All data were concluded from at least three independent assays. Statistic data were displayed as mean ± SEM and were conducted unpaired *t* test analysis or one-way ANOVA analysis to determine significant difference. * *p* < 0.05, ** *p* < 0.01, *** *p* < 0.001 compared with the negative group.

Given the effect of IC87114, we then designed p110δ-knockdown macrophages for further study. Three different siRNAs targeting mouse p110δ were transfected and macrophages with the minimal level of p110δ were screened (Fig. S2A). Next, we conducted ELISA assay and found that TiPs-induced TNFα and IL-6 secretions from p110δ-knockdown macrophages were markedly suppressed (Fig. 3A). However, TNFα and IL-6 mRNA expressions were almost unchanged upon reduced p110δ (Fig. 3B). These results were aligned with the effect of inhibition of p110δ.

Next, Brefeldin A (BFA), an inhibitor to hinder the protein trafficking from Endoplasmic Reticulum (ER) to Golgi complex, was applied for WB assay. After macrophages were co-treated with BFA and TiPs, we found that protein levels of TNFα and IL-6 were unaffected upon p110δ-knockdown, suggesting that p110δ was responsible for post-trafficking process of TNFα and IL-6 (Fig. 3C). For further validation, IF staining depicted less surface TNFα staining but stronger staining around the nucleus, remarkably co-localizing with TGN38 which labeled trans-Golgi network in p110δ-knockdown macrophages (Fig. 3D, E). Aligned with that, IF staining for IL-6 also exhibited more scattering distribution in si-NC macrophages (Fig. 3F). According to FCM assay, knockdown p110δ strikingly declined surface distribution of TNFα from 52.88 ± 3.63% to 19.92 ± 6.24% while intracellular TNFα was elevated (Figs. 3G and S2B). In conclusion, these results further confirmed that knockdown p110δ alleviated the TiPs-induced TNFα and IL-6 secretions by disturbing their trafficking from Golgi complex to plasma membrane.

Next, we wondered whether extra p110δ could facilitate TNFα and IL-6 secretions. Intriguingly, we generated p110δ-overexpressed macrophages and found that the overexpression of p110δ merely affected the serum levels of TNFα and IL-6 (Fig. S2C–E). Similarly, FCM assay implied that under 2 h TiPs treatment, elevated p110δ barely increased the surface level of TNFα and the overexpression of p110δ had no impact on the TNFα and IL-6 expressions (Fig. S2F–J).

Next, downstream factors and signaling pathways of p110δ were also further explored. We performed WB assay and found comparable activation of NF-κB and MAPK signaling pathways upon different levels of p110δ (Fig. 3H, I). It was reported that LPS resulted in the co-localization of dynamin 2 (Dyn2) and P230 (a specific label of the tubules, which is responsible for transporting TNFα as cargo), and the co-localization could be abrogated by IC87114 [17, 32]. Therefore, we treated TiPs-stimulated macrophages with IC87114 for 2 h and found that the inhibition of p110δ quenched the co-localization between Dyn2 and P230 (Fig. S3A). Apart from that, dynasore, a dynamin GTPase inhibitor, could drastically impaired the TiPs-induced TNFα and IL-6 secretions (Fig. S3B). AKT, as another PI3K downstream factor, was widely reported responsible to various proteins trafficking to plasma membrane [33–35]. Here, confocal microscopy also portrayed a co-localization between p-AKT and P230 upon TiPs stimulation, which was abrogated by IC87114 (Fig. S3C). GSK690693, functioned as a pan-AKT inhibitor, could impaire TNFα and IL-6 secretions upon 8 h TiPs stimulation (Fig. S3D). These data implied that Dyn2 and AKT might be the vital downstream players for p110δ.

### Pik3cd transcription was mediated by KLF4 in TiPs-stimulated macrophages

**Fig. 4:**
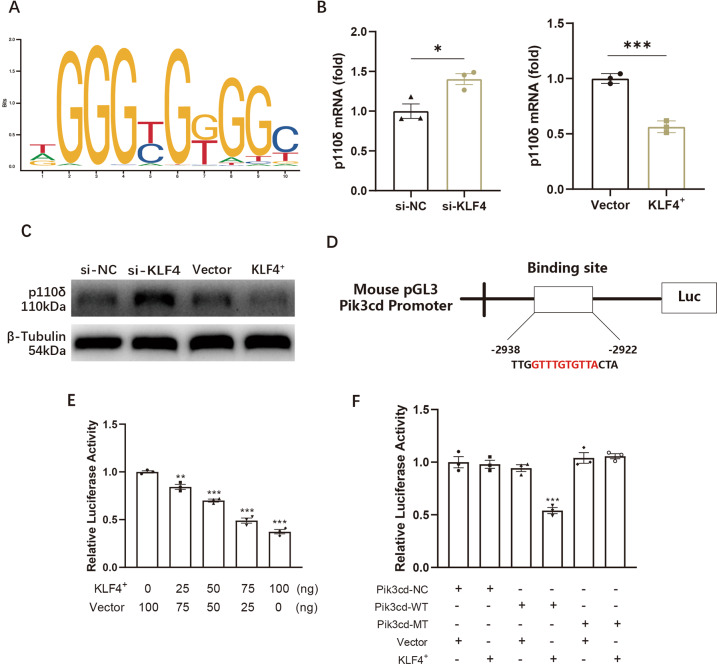
Pik3cd transcription was mediated by KLF4 in TiPs-stimulated macrophages. **A** Predicted sequence logo of KLF4 binding site was acquired by JASPAR database. **B** mRNA expression of p110δ from KLF4-knockdown RAW264.7 or KLF4-overexpressed RAW264.7 was detected. **C** Protein level of p110 from KLF4-knockdown RAW264.7 or KLF4-overexpressed RAW264.7 was detected. **D** Structure of plasmids containing mutant-type of Pik3cd promoter for luciferase reporter assay. **E** Relative luciferase activity upon different supplement of KLF4-overexpressed (KLF4^+^) plasmids. **F** Relative luciferase activity was analysed after transfecting plasmids containing wild-type (Pik3cd-WT) or mutant-type (Pik3cd-MT) of Pik3cd promoter with or without KLF4^+^ plasmids. All data were concluded from at least three independent assays. Statistic data were displayed as mean ± SEM and were conducted unpaired *t* test analysis or one-way ANOVA analysis to determine significant difference. *p < 0.05, **p < 0.01, ***p < 0.001 compared with the negative group.

We next aimed at exploring the upstream regulator of p110δ. Referring to the Gene database of The National Center for Biotechnology Information (NCBI) (https://www.ncbi.nlm.nih.gov/), the sequence of Pik3cd (gene ID of p110δ) was acquired. In all, 2000 bp upstream and 100 bp downstream of the transcriptional initiated site was considered as the binding realm for transcription regulators. Next, UCSC (http://www.genome.ucsc.edu/) and JASPAR (http://jaspar.genereg.net/) were utilized to sift the underlying transcription factors, among which KLF4 with the highest score was selected. (Fig. 4A).

Next, we applied siRNA and plasmids to construct the most effectively KLF4-knockdown (si-KLF4) or KLF4-overexpressed (KLF4^+^) macrophages (Fig. S4A, B). We next conducted WB and qPCR assays, which suggested that knockdown KLF4 enhanced the expression of p110δ while overexpressed KLF4 resulted in decreased level of p110δ (Fig. 4B, C). For further validation, plasmids carrying wild-type of Pik3cd-promoter (Pik3cd-WT) or mutant-type Pik3cd-promoter (Pik3cd-MT) were designed for dual luciferase reporter assay (Fig. 4D). With the increasing dosage of KLF4^+^ plasmids were added for co-transfection, fluorescence magnitude was declined (Fig. 4E). Next, Pik3cd-WT plasmids or Pik3cd-MT plasmids together with KLF4^+^or vector plasmids were co-transfected and weaken luciferase activity was obtained upon the transfection of Pik3cd-WT and KLF4^+^ (Fig. 4F). These results suggested that KLF4 functioned as a transcriptional repressor for Pik3cd.

### Overexpressed KLF4 attenuated TiPs-induced inflammation by suppressing NF-κB pathway and macrophages M1/M2 polarization

**Fig. 5:**
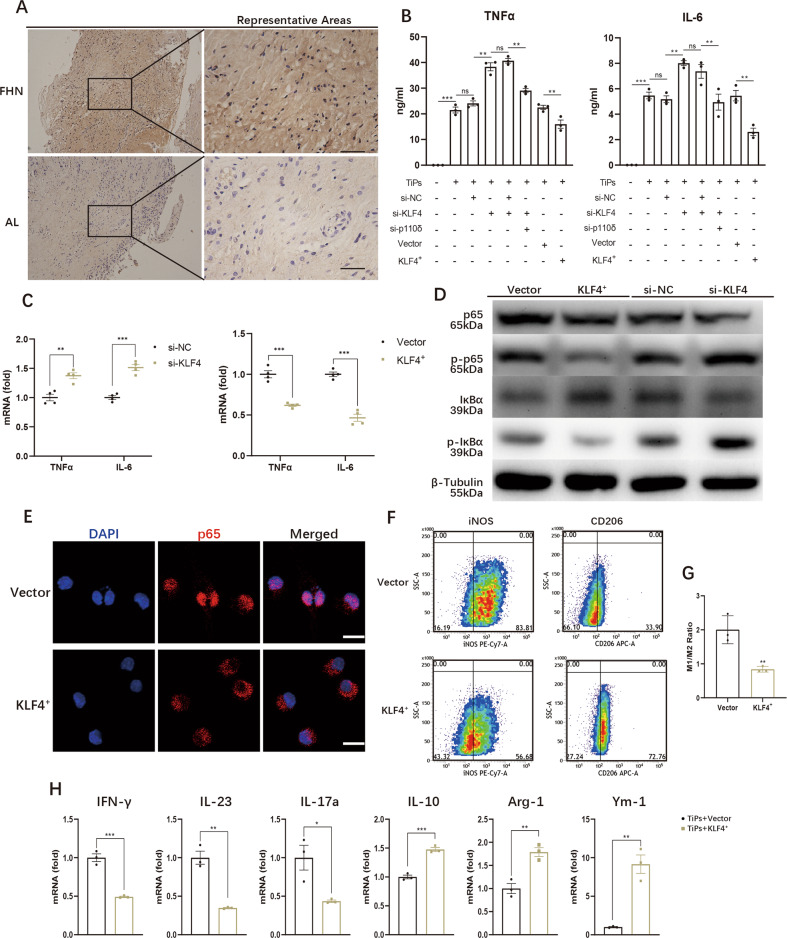
Overexpressed KLF4 attenuated TiPs-induced inflammation by suppressing NF-κB pathway and macrophages M1/M2 polarization. **A** IHC staining result of human synovial membranes from femoral head necrosis (FHN) patients and prosthetic aseptic loosening (AL) patients to detect the expression of KLF4. The black bar, 50 μm. **B** Supernatant TNFα and IL-6 from KLF4-knockdown (si-KLF4) or KLF4-overexpressed (KLF4^+^) macrophages were analysed by ELISA assay after 8 h TiPs stimulation. **C** mRNA expressions of TNFα and IL-6 in KLF4-knockdown macrophages or KLF4-overexpressed macrophages were examined after 8 h TiPs activation. **D** Total p65, total IκBα, phosphorylated p65 and phosphorylated IκBα were detected by WB assay to determine the activation of NF-κB signaling pathway upon 1 h TiPs stimulation in KLF4-knockdown macrophages or KLF4-overexpressed macrophages. **E** After 1 h TiPs stimulation, nuclear translocation of p65 was observed by confocal microscopy in Vector or KLF4-overexpressed macrophages. The white bar, 10 μm. **F** Macrophage polarization upon 24 h TiPs stimulation was estimated by FCM assay with iNOS symbolizing M1 polarization and CD206 symbolizing M2 polarization. **G** Quantitative analysis of FCM assay to measure macrophages M1/M2 ratio in Vector or KLF4-overexpressed macrophages upon 24 h TiPs stimulation. **H** The expressions of macrophage M1 polarization relative genes (IFN-γ, IL-23, IL-17a) and M2 polarization relative genes (IL-10, Arg-1, Ym-1) were detected by qPCR assay. All data were concluded from at least three independent assays. Statistic data were displayed as mean ± SEM and were conducted unpaired *t* test analysis or one-way ANOVA analysis to determine significant difference. *p < 0.05, **p < 0.01, ***p < 0.001 compared with the negative group.

Next, we further explored the concrete role of KLF4 in TiPs-induced inflammation. Firstly, we performed synovial membrane IHC staining and it portrayed weaken KLF4 staining in AL group with an average IS of 1.26 ± 0.06 (average IS of FHN group was 2.25 ± 0.07, *p* < 0.05) (Figs. 5A and S4C), implying an anti-inflammatory role of KLF4. Then ELISA assay was performed with results showing that knockdown KLF4 aggravated the TNFα and IL-6 secretions while overexpression of KLF4 engendered lower serum levels of TNFα and IL-6 (Fig. 5B). Sequentially, TNFα and IL-6 mRNA expressions were also detected, indicating that KLF4 level was negatively relevant with TNFα and IL-6 expressions (Fig. 5C). This outcome was inconsistent with the role of p110δ, implying that there might be other downstream pathways for KLF4 to alleviated TiPs-induced macrophages inflammation.

Thus, we performed WB assay and we discovered that upon 1 h TiPs stimulation, knockdown KLF4 facilitated the phosphorylation of p65 and IκBα while total IκBα was reduced, but overexpressed KLF4 led to contrary outcome (Fig. 5D). Furthermore, IF assay was performed, which indicated that TiPs-induced p65 translocation was suppressed upon increased level of KLF4 (Fig. 5E). Next, macrophages polarization was detected by FCM and qPCR assay, and we found that overexpressed KLF4 contributed to descendant M1/M2 ratio from 2.01 ± 0.24 to 0.84 ± 0.05, with reduced expressions of M1 relative factors (IFN-γ, IL-23, IL-17a) and increased expressions of M2 relative factors (IL-10, Arg-1, Ym-1) (Figs. 5F–H and S4D). Taken together, KLF4 could inhibit TiPs-induced inflammation by mediating NF-κB pathway and M1/M2 polarization.

### MiR-92a elevated the expression of p110δ and promoted TiPs-induced inflammation by targeting KLF4

**Fig. 6:**
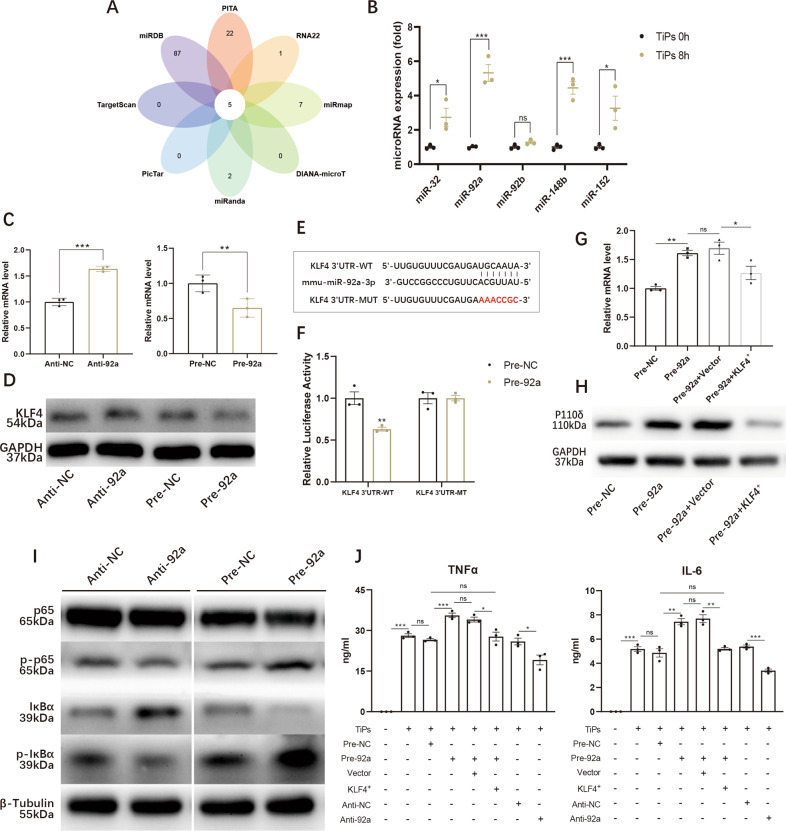
MiR-92a elevated the expression of p110δ and promoted TiPs-induced inflammation by targeting KLF4. **A** Potential microRNAs interacting with KLF4 were sorted out from online platform The Encyclopedia of RNA Interactomes (ENCORI) and microRNAs were categorized by Venn chart. **B** Expressions of potential microRNAs upon 8 h TiPs stimulation were detected by qPCR assay. **C**, **D** mRNA (**C**) and protein levels (**D**) of KLF4 were respectively examined upon the transfection of mimics-miR-92a (Pre-92a) or inhibitor-miR-92a (Anti-92a). **E** Potential binding region of miR-92a and 3′UTR of KLF4, and relative sequence of KLF4 with mutant 3′UTR. **F** Dual luciferase activity was detected after 48 h transfection of wild-type KLF4 3′UTR (KLF4 3′UTR-WT) or mutant-type KLF4 3′UTR (KLF4 3′UTR-MT) with mimics-miR-92a (Pre-92a) or mimics-NC (Pre-NC). **G**, **H** mRNA level (**G**) and protein levels (**H**) of p110δ were respectively examined upon the transfection of mimics-miR-92a (Pre-92a) with or without KLF4-overexpressed plasmids (KLF4^+^). **I** Total p65, total IκBα, phosphorylated p65, and phosphorylated IκBα were detected by WB assay to determine the activation of NF-κB signaling pathway upon 1 h TiPs stimulation in mimics-miR-92a (Pre-92a)-transfected macrophages or inhibitor-miR-92a (Anti-92a)-transfected macrophages. **J** Supernatant levels of TNFα and IL-6 from miR-92a-increased or miR-92a-inhibited macrophages were analysed by ELISA assay after 8 h TiPs stimulation. All data were concluded from at least three independent assays. Statistic data were displayed as mean ± SEM and were conducted unpaired t test analysis or one-way ANOVA analysis to determine significant difference. *p < 0.05, **p < 0.01, ***p < 0.001 compared with the negative group.

Reportedly, KLF4 could be targeted by various microRNAs [28–30]. Therefore, we next explored if there was a microRNA interacting with KLF4 and involving in TiPs-induced inflammation. Online platform The Encyclopedia of RNA Interactomes (ENCORI) was utilized to acquire the data from PITA, RNA22, miRmap, DIANA-microT, miRanda, PicTar, TargetScan, miRDB. After categorized by Venn chart, five microRNAs potentially targeting KLF4 were included (Fig. 6A). After treated with TiPs, macrophages were conducted qPCR assay to explore the expression of microRNAs, among which the expression of mmu-miR-92a-3p (miR-92a) achieved the most predominant facilitation (Fig. 6B).

Next, we generated mimics-miR-92a (Pre-92a) or inhibitor-miR-92a (Anti-92a) transfected macrophages for gain- or loss- function experiments. As demonstrated in WB and qPCR assays, increased miR-92a obviously reduced the expression of KLF4 in both protein and mRNA levels, while impaired miR-92a exhibited reverse effect (Fig. 6C, D). To ensure the relationship between miR-92a and KLF4, we acquired the complementary sequence between miR-92a and KLF4 from TargetScan database and constructed firefly luciferase reporter plasmids containing mutant type of KLF4 3′UTR (KLF4 3′UTR-MT) or wild-type KLF4 3′UTR (KLF4 3′UTR-WT) (Fig. 6E). Compared with KLF4 3′UTR-MT, KLF4 3′UTR-WT resulted in impaired luciferase activity when co-transfected with Pre-92a, validating that KLF4 was the target of miR-92a (Fig. 6F). Next, Pre-92a was transfected with or without KLF4+, and it demonstrated that the expression of p110δ, which was strengthened by Pre-92a, reduced upon the co-transfection of Pre-92a and KLF4^+^, implying miR-92a could facilitate p110δ expression by targeting KLF4 (Fig. 6G, H). Considering the interaction of KLF4 and NF-κB signaling pathway, WB assay was then conducted and we found that Pre-92 was positively relative to the activation of NF-κB signaling pathway (Fig. 6I). Meanwhile, the serum levels of TNFα and IL-6 were detected and results indicated that Pre-92a contributed to exacerbated the secretions of TNFα and IL-6 while Anti-92a alleviated their secretions. Co-transfected with KLF4^+^, the Pre-92a magnified TNFα and IL-6 secretions were mitigated (Fig. 6J). Taken together, we suggested miR-92a as an upstream mediator for KLF4-p110δ interplay and miR-92 could also amplify the TiPs-induced inflammation by activating NF-κB signaling pathway.

### Knockdown KLF4 exacerbated TiPs-induced osteolysis which was attenuated by knockdown p110δ

**Fig. 7:**
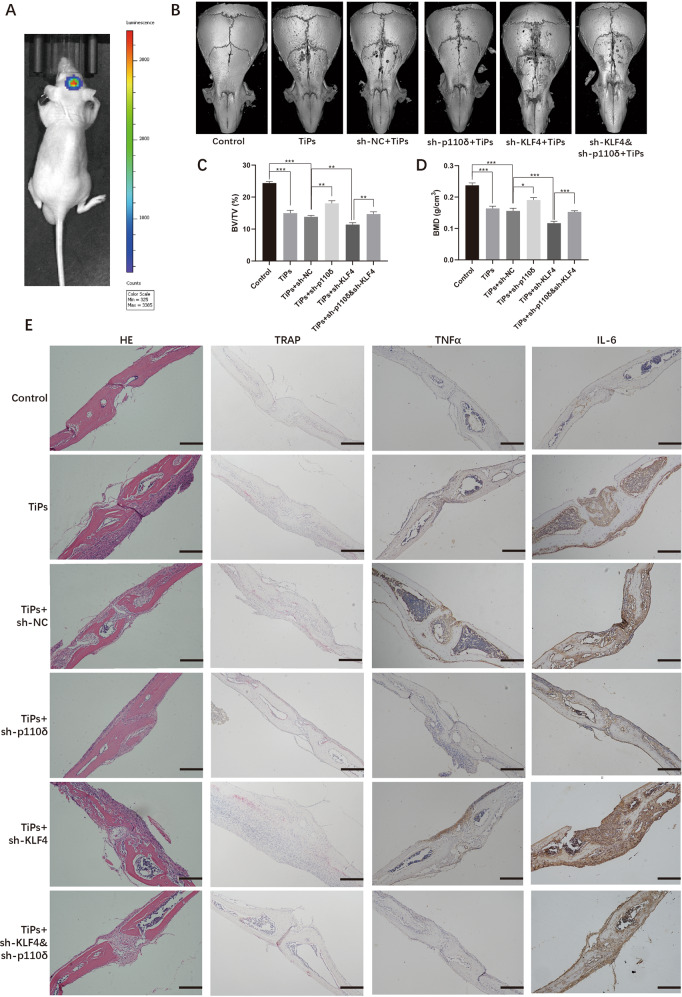
Knockdown KLF4 exacerbated TiPs-induced osteolysis which was attenuated by knockdown p110δ. **A** Noticeable bioluminescence signal from calvarias was detected 7 days after surgery. **B** 3D construction was implemented and representative images of calvarias from each group were listed after micro-CT imaging. **C**, **D** Bone mineral density (BMD) and the ratio of bone volume against total volume (BV/TV) from each group were calculated after 3D construction. **E** Sections loading calvarias from each group were performed H&E staining, TRAP staining and IHC staining of TNFα and IL-6. The black bar, 200 μm. Statistic data were displayed as mean ± SEM and were conducted one-way ANOVA analysis to determine significant difference. *p < 0.05, **p < 0.01, ***p < 0.001 compared with the negative group.

After revealing the role of p110δ and KLF4 in TiPs caused macrophages inflammation, we generated nude mice cranial osteolysis model according to the previous researches for vivo experiments [31, 36], and grouping was described in *Methods* section. At day 7 after surgery, we could detect strong luciferase signal, which meant that macrophages remained on the calvaria. (Fig. 7A). Then, calvarias were gathered for micro-CT scanning. We found that sh-p110δ transfection apparently attenuated the TiPs caused osteolysis with higher BMD and BV/TV ratio, while sh-KLF4 transfection induced opposite consequence (Figs. 7B–D and S5A). Next, sections loading calvarias were performed TRAP staining and we noticed that TiPs+sh-p110δ group exhibited less osteoclasts (22.33 ± 1.20 vs 34.83 ± 1.20, *p* < 0.05) while more osteoclasts were detected in TiPs+sh-KLF4 group (41.17 ± 1.52 vs 34.83 ± 1.20, *p* < 0.05) (Figs. 7E and S5B). TNFα and IL-6 IHC staining results showed that abrogated p110δ resulted in slighter staining of TNFα and IL-6 than those in sh-KLF4 group (*p* < 0.05) (Figs. 7E and S5C, D). In conclusion, these results suggested p110δ as a pro-osteolysis factor while KLF4 was an anti-osteolysis factor.

## Discussion

In this study, we revealed the pro-inflammatory role of p110δ, which was mediated by miR-92a/KLF4 in TiPs-stimulated macrophages. Firstly, we screened p110δ as a TiPs-induced inflammation relative factor and confirmed that its expression was facilitated toward TiPs stimulation. With the application of IC87114 or si-p110δ, we found that p110δ involved in the transport of TNFα and IL-6 from Golgi complex to plasma membrane. KLF4, targeted by miR-92a, was predicted as a transcriptional repressor of p110δ and it could also attenuate the TiPs activated NF-κB pathway and M1/M2 macrophages polarization ratio. In vivo experiments, the role of KLF4 and p110δ were further confirmed in TiPs-induced mice cranial osteolysis, suggesting p110δ as a pro-osteolysis factor while KLF4 was an anti-osteolysis factor.

Ubiquitous reports indicated that wear particles induced prosthetic aseptic loosening was initiated by a series of pro-inflammatory factors from macrophages [37–39]. Our study also confirmed that levels of TNFα and IL-6 were elevated in synovial membranes from prosthetic aseptic loosening patients and in TiPs-treated macrophages. According to online database and the RNA-seq analysis we formerly conducted, we assumed p110δ might also involve in TiPs-induced inflammation and further assays were performed to unravel the role of p110δ.

After we discovered that the inhibition or knockdown of p110δ could not impaired the mRNA expression of TNFα and IL-6, we assumed that p110δ functioned in the post-transcriptional modification process or protein trafficking of TNFα and IL-6. Next, p110δ-knockdown macrophages were constructed and BFA was applied to block protein trafficking from Endoplasmic Reticulum (ER) to Golgi complex for WB assay. If p110δ mediated the post-transcriptional modification process, the knockdown level of p110δ would lead to reduced TNFα and IL-6 protein levels in BFA-treated macrophages. Else, the knockdown level of p110δ would not affect the protein levels of TNFα and IL-6 since the treatment of BFA had already abrogated their transport from ER to Golgi complex. As a result, comparable protein levels of TNFα and IL-6 were detected between p110δ-knockdown group and control group after 8 h BFA and TiPs treatment, validating that p110δ was responsible for TNFα and IL-6 transport, and this point of view was further confirmed by IF staining and FCM assay.

Intriguingly, we also found that p110δ overexpression could not promoted TNFα and IL-6 secretions in TiPs-stimulated macrophages. This reminded us that the trafficking was a multi factors-controlled process and the overexpressed p110δ might not be enough to facilitated the protein transport. On the other hand, whether the trafficking process was the bottleneck of inflammatory cytokines secretion was under discussion, and some argued that the accumulation of trafficking protein was temporary. Rather than transport, synthesis was the rate-limit step [40]. And there were reports indicating the accumulation of TNFα and IL-6 in Golgi complex was temporary [41–43]. Therefore, we also presumed that the elevated expression of p110δ induced by TiPs stimulation might be enough for TNFα and IL-6 trafficking, and extra p110δ would not further accelerate their secretion. Instead, the synthesis of TNFα and IL-6 might be the prominent rate-limiting step for TNFα and IL-6 secretion.

After we found that KLF4 was the transcription repressor of p110δ, further role of KLF4 was confirmed, and our findings were aligned with the previous reports that KLF4 was an anti-inflammatory factor [44–47]. Under the generation of mice cranial osteolysis model, the role of KLF4/ p110δ was further confirmed, indicating KLF4/p110δ as promising targets for TiPs-induced osteolysis.

## Materials and methods

### Human synovial membranes collection

Approved by the ethics committee, synovial membranes of 15 patients, including 8 femoral head necrosis (FHN) patients (three males and five females) and 7 hip prosthetic aseptic loosening (AL) patients (three males and four females), from Department of Orthopaedic Surgery, Sun Yat-sen Memorial Hospital, Sun Yat-sen University were collected following the guidelines for human care. After 1 day fixation in 4% paraformaldehyde, specimens were decalcified in EDTA decalcifying solution (E1171, Solarbio). Sealed in paraffin, synovium was made into section for IHC assays. Specific information of subjects was mentioned in our previous study [31]. All patients have consented the collection and the usage of the samples, and experiments were approved by the ethics committee at the Sun Yat-sen University, Sun Yat-sen Memorial Hospital (2017 ethic record no: 26).

### Antibodies, inhibitors, plasmids, and lentiviruses

Rabbit antibody against phospho-SAPK/JNK (#4668, for WB assay), phospho-p44/42 MAPK (#4370, for WB assay), phospho-p38 MAPK (#4511, for WB assay), GAPDH (#5174, for WB assay), phospho-NF-κB p65 (#3033, for WB assay), NF-κB p65 (#8242, for WB and IF assays), phospho-IKKα/β (#2694, for WB assay), IKKβ (#8943, for WB assay), phospho-IκBα (#5209, for WB assay), and IκBα (#4812, for WB assay) were purchased from Cell Signaling Technology (Massachusetts, USA). Rabbit antibody against PI3Kinase p110 delta (ab109006 for WB and IF assays, ab200372 for IHC assay) was obtained from Abcam (Cambridge, UK). PE-Cyanine7-conjugated TNF alpha Monoclonal Antibody (MP6-XT22, for FCM assay), PE-Cyanine7 conjugated iNOS monoclonal antibody (25-5920-80, for FCM assay), APC conjugated CD206 monoclonal antibody (17-2061-82, for FCM assay), mouse antibody against TGN38 monoclonal antibody (MA3-063, for IF assay) was purchased from ThermoFisher Scientific (Massachusetts, USA). Rabbit antibody against β-tubulin (A17913, for WB assay), KLF4 (A13673, for FCM assay), P230 (A10216, for IF assay), TNF-α (A11543, for WB, IF and IHC assay), IL-6(A0286, for IF and IHC assay), and IL-6 (A11114, for WB assay) was purchased from ABclonal (Wuhan, China). Mouse antibody against Dyn2 (sc-166669, for IF assay) was purchased from Santa Cruz Biotechnology (Texas, USA). p110δ inhibitor (IC87114, S1268), Dyn2 inhibitor (Dynasore, S8047), AKT inhibitor (GSK690693, S1113), and TAPI-1 (S7434) was purchased from Selleck (Texas, USA). Rabbit antibody against IL-6 (21865-1-AP, for IHC assay) was purchased from Proteintech (Chicago, USA). LPS was purchase from Sigma-Aldrich (L2880, Missouri, USA).

To generated gene-altered macrophages, siRNA, plasmids or recombinant lentiviruses were obtained from GenePharma (Shanghai, China). SiRNAs targeting p110δ (GeneID: 18707)/KLF4 (GeneID: 16600) were constructed and denoted as si-p110δ and si-KLF4 respectively. Si-NC was taken as negative control. P110δ/KLF4 overexpressed plasmids were constructed and denoted as p110δ^+^ and KLF4^+^ respectively. Plasmids named Vector were taken as negative control. Recombinant lentiviruses were constructed to stably knockdown p110δ/KLF4 or to stably overexpress p110δ/KLF4, denoting as sh-p110δ/sh-KLF4 and OE-p110δ/OE-KLF4 respectively, with lentiviruses named sh-NC or OE-vector as negative control. To generated miR-92a-altered macrophages, mimics-miR-92a (Pre-92a) and relative negative-control microRNAs (Pre-NC), inhibitor-miR-92a (Anti-92a) and relative negative-control microRNAs (Anti-NC) were obtained from RiboBio. The lentivirus expressing firefly luciferase (fluc-lentivirus) from OBiO Technology (Shanghai, China) was applied for the construction of bioluminescent reporter cells to detect the bioluminescence signal.

### Preparation of titanium particles

The procedure to generate sterilized TiPs was described in our previous study [31]. Briefly, TiPs (<20 μm, W08A030, Alfa Aesar) were sifted until averaging 0.82 ± 0.12 μm and were washed by 75% ethanol. Sterilized by firing at 180 °C for 6 h, TiPs were obtained with endotoxin level <0.25 EU ml^−1^ (Limulus assay, EC80545, Bioendo). Processed TiPs were then diluted with PBS into the concentration of 1.8 × 10^−3^g/mL for experiments.

### Cell culture and transfection

Under 37 °C and 5% CO_2_ circumstance, RAW264.7 (CL-0190, Procell) were commonly cultivated in the growth medium-high glucose Dulbecco’s modified Eagle’s medium (DMEM) (C11995500BT, ThermoFisher Scientific) containing 10% fetal bovine serum (FBS) (10099141, ThermoFisher Scientific) and 1% penicillin-streptomycin solution (SV30010, ThermoFisher Scientific). Before stimulation, cells were seeded in plates for 24 h until 70–80% confluence.

As for transient transfection, Lipofectamine RNAiMAX Reagent (13778075, ThermoFisher Scientific) and plasmids were diluted and mixed as a system by Opti-MEM Medium (31985088, ThermoFisher Scientific) for 24–48 h transfection according to the specification. Similarly, miR-92a mimics or miR-92a inhibitor were transfected with the assistance of Lipofectamine 3000 (L3000015, ThermoFisher Scientific). As for stable transfection, 1 × 10^7^ transducing unit (TU) per mL lentiviruses contained in cultured medium was applied for 24–48 h transfection.

### Cell viability assay

After seeded in 96-well plate in the concentration of 5 × 10^3^ for 24 h, TiPs with or without IC87114 were applied for stimulation for 16 h. Sequentially, Cell Counting Kit-8 (K1018, ApexBio Technology) was utilized according to the specification. After 37°C incubation for 2 h, cell viability was detected by gauging the absorbance at 450 nm.

### Qualitive real-time PCR assay

Total RNA was extracted by TRIzol^TM^ Reagent (15596026, ThermoFisher Scientific) and was sequentially purified by chloroform, isopropyl alcohol, ethanol, 75% ethanol and RNase-Free Water. RNA concentration was measured by NanoDrop 2000 (ND-2000, ThermoFisher Scientific). Then, RNA solution was blended with PrimeScript RT Master Mix (RR036D, TaKaRa Biotechnology) and according to the specification, reverse transcription was conducted. After obtaining cDNA solution, UNICONTM qPCR SYBR Green Master Mix (11198ES08, Yeasen), specific primers and RNase-Free Water were applied for qualitive real-time PCR by Roche LightCycler 96 Real-Time PCR system (Roche Molecular Systems, Inc.). The primers of the target genes were listed in Table 1.

**Table 1:** Primers for cDNA synthesis or qPCR detection of RT-qPCR.

Primer names	Sequences
TNFα	F: CAGGCGGTGCCTATGTCTC
	R: CGATCACCCCGAAGTTCAGTAG
IL-6	F: GAAATGCCACCTTTTGACAGTG
	R: TGGATGCTCTCATCAGGACAG
p110δ	F: GTAAACGACTTCCGCACTAAGA
	R: GCTGACACGCAATAAGCCG
KLF4	F: AGGAACTCTCTCACATGAAGCG
	R: GGTCGTTGAACTCCTCGGTC
GAPDH	F: TGTGTCCGTCGTGGATCTGA
	R: TTGCTGTTGAAGTCGCAGGAG
mmu-miR-32-5p	F: GCGGGCTATTGCACATTACTA
	R: GGTCGTTGAACTCCTCGGTC
	RT: GTCGTATCCAGTGCAGGGTCCGAG
	GTATTCGCACTGGATACGACTGCAACT
mmu-miR-148b-3p	F: GCGGGCTCAGTGCATCACAGA
	R: GCAGGGTCCGAGGTATTC
	RT: GTCGTATCCAGTGCAGGGTCCGAG
	GTATTCGCACTGGATACGACACAAAGT
mmu-miR-92a-3p	F: GCGGGCTATTGCACTTGTCCC
	R: GCAGGGTCCGAGGTATTC
	RT: GTCGTATCCAGTGCAGGGTCCGAG
	GTATTCGCACTGGATACGACCAGGCCG
mmu-miR-92b-3p	F: GCGGGCTATTGCACTCGTCCC
	R: GCAGGGTCCGAGGTATTC
	RT: GTCGTATCCAGTGCAGGGTCCGAG
	GTATTCGCACTGGATACGACGGAGGCC
mmu-miR-152-3p	F: GCGGGCTCAGTGCATGACAGA
	R: GCAGGGTCCGAGGTATTC
	RT: GTCGTATCCAGTGCAGGGTCCGAG
	GTATTCGCACTGGATACGACCCAAGTT
U6	F: TGCGGGTGCTCGCTTCGGCAGC
	R: CCAGTGCAGGGTCCGAGGT
	RT: GTCGTATCCAGTGCAGGGTCCGAGG
	TGCACTGGATACGACAAAATATGGAAC

*F* forward primers designed for the qPCR step of RT-qPCR, *R* reverse primers designed for the qPCR step of RT-qPCR, *RT* primers for cDNA synthesis step of RT-qPCR.

### Western blot assay

Lysate solution consisted of RIPA buffer (#9806 S, Cell Signaling Technology), PMSF (P0100-1, Solarbio), and phosphatase inhibitor cocktail (CW2383, CW Biotech) was applied for protein extraction. After centrifugation, the protein concentration was gauged and normalized by Bicinchoninic acid (BCA) protein assay (23225, ThermoFisher Scientific). Separated and transferred to the polyvinylidene fluoride (PVDF) membranes (3010040001, Sigma-Aldrich), protein was blocked with 5% Bovine Serum Albumin (BSA) (ST023, Beyotime). Overnight incubation of primary antibodies at 4°C and 1 h incubation of HRP-linked secondary antibody at room temperature were sequentially conducted. With the assistance of Super ECL Detection Reagent (36208ES60, Yeasen), immunoblotting results were obtained with digital imaging system (Syngen G:BOX Chmi XT4).

### Assaying cytokine secretion

To estimate serum levels of TNFα and IL-6, supernatant was collected and purified by centrifugation. Referring to specification, instant enzyme-linked immunosorbent assay (ELISA) was operated with specific kit (EMC102a.96 for TNFα analysis and EMC004.96 for IL-6 analysis, Neobioscience).

### Immunofluorescence staining

Before stimulation, 5 × 10^4^ macrophages were cultivated in a confocal dish for 24 h (BDD011035, Jet Bio-Filtration Co., Ltd). After stimulation, 4% paraformaldehyde were applied for fixation. For intracellular TNFα and IL-6 detection, 0.1% Triton X-100 was implemented for permeabilization. As for surface TNFα detection, 10 μM TAPI-1 was applied to inhibit TNFα secretion and cells were carried out IF staining without permeabilization. Blocked with 1% BSA for 30 min, cells were sequentially incubated with primary and secondary antibodies, followed by DAPI (HNFD-02, HelixGen Co., Ltd) for antifade fluorescence disposal. Confocal microscopy (LSM 710, Carl Zeiss) was operated for imaging and further analysis was carried out by ZEN 2011 (blue edition) software (version 1.0).

### Flow cytometry assay

Cells were harvested by 0.25% Trypsin-EDTA (25200056, ThermoFisher Scientific) and were washed with PBS. Then, fixation was performed with Fixation Medium (Medium A) (GAS001S100, ThermoFisher Scientific) and Permeabilization Medium (Medium B) (GAS002S5, ThermoFisher Scientific) was applied for permeabilization to detect intracellular TNFα, iNOS or CD206. Blocked with 1% BSA for 30 min, cells were incubated with PE-Cyanine7-conjugated TNFα antibody, or PE-Cyanine7-conjugated iNOS antibody, or APC conjugated CD206 antibody for 20 min. Further analysis was conducted with Flow Cytometry Instruments (BD FACSVerse, BDBiosciences).

### Dual-luciferase reporter assay

In order to confirm the relation between p110δ and KLF4, HEK-293T cells (CL-0005, Procell) were seeded in 24-well plates and after 24 h, cells reached a confluence of 70%-80%. According to the manufacturer’s specifications of Dual-Glo Luciferase Assay System, Vector/KLF4^+^ plasmids together with plasmids loading wild-type Pik3cd promoter (Pik3cd-WT) or mutant-type (Pik3cd-MT) Pik3cd promoter were transfected. After 48 h, luciferase activity was detected with Firefly/Renilla Luciferase Assay Reagent by SYNERGY H1 (Bio-Tek). pRL-TK plasmids expressing Renilla luciferase were applied for collation. Similarly, Pre-NC/Pre-92a and plasmids containing wild type (KLF4 3′UTR-WT) or mutant type 3′‐UTR (KLF4 3′UTR-MT) of KLF4 were transfected into HEK-293T for exploring the relationship between miR-92a and KLF4.

### Animal surgery

In order to construct the mice cranial osteolysis model, male BALB/c nude mice aging 10 weeks (Animal Laboratory of Sun Yat-sen University) were acquired for vivo experiments. After anesthesia, a 10-mm midline sagittal incision was operated to uncover calvarias and 0.5 × 0.5 × 0.5 cm^3^ gelatin sponges were employed as retainers for injection. Divided into six groups with six mice for each, mice were treated with different injections as followed: (1) 100 μl PBS was applied in Control group; (2)100 μl PBS with 3 mg TiPs was applied in TiPs group; (3) 100 μl PBS with 0.5 × 10^6^ NC-stably knockdown RAW264.7 and 3 mg TiPs were applied in TiPs+sh-NC group; (4) 100 μl PBS with 0.5 × 10^6^ p110δ stably-knockdown RAW264.7 and 3 mg TiPs were applied in TiPs+sh-p110δ group; (5) 100 μl PBS with 0.5 × 10^6^ KLF4 stably-knockdown RAW264.7 and 3 mg TiPs were applied in TiPs+sh-KLF4 group; (6) 100 μl PBS with 0.5 × 10^6^ KLF4 and p110δ stably-knockdown RAW264.7 and 3 mg TiPs were applied in TiPs+sh-KLF4&sh-p110δ group. Apart from that, a bioluminescence group was applied: 100 μl PBS with 0.5 × 10^6^ bioluminescent reporter cells were injected and 7 days after surgery, bioluminescence signal were detected. A week after the procedure, mice calvarias were isolated for micro-CT imaging or IHC assay. No blinding was applied in the procedure and mice were randomly divided into six groups. All the animal procedures were performed under the guidelines for the care and use of laboratory animals of Sun Yat-sen University and were approved by the Animal Ethical and Welfare Committee of Sun Yat-sen University Cancer Center (L102042020120Q).

### Immunohistochemistry assay

Calvarias or human synovial membranes loaded by sections were embedded in paraffin. Heated at 60 °C for 2 h, the sections were sequentially treated by xylene, ethanol, 90% ethanol, 80% ethanol, 70% ethanol, 60% ethanol and PBS for deparaffinization and rehydration. Then pepsin (ZLI-9013, ZSGB-BIO) was applied to restore antigenicity for 20 min and 25 min 3% H_2_O_2_ incubation was utilized to quench endogenous peroxidaseactivity. After incubated in 3% bovine serum albumin (BSA) (CCS30014.01, MolecularResearch Center, Inc.) for 30 min, sections were next incubated in diluted primary antibody followed by incubation of HRP conjugated Goat Anti-Rabbit IgG H&L antibody (ab6721, Abcam). DAB Horseradish Peroxidase Color Development Kit (P0203, Beyotime) was applied for visualization. Nuclear staining was processed with hematoxylin and sections were eventually sealed off by neutral balsam (G8590, Solarbio). Biomicroscope (Nikon eclipse 80i, Japan) was applied for observation with Bresalier’s analysis for quantitative analysis. As for Bresalier’s analysis, 10 fields of each section were randomly chosen to estimate the staining score (score 0: stainless; score 1: slightly staining; score 2: moderate staining; score 3: strong staining) and the scale of each staining level (Fx). Eventually, average intensity score (IS) was calculated as follows: ∑(0 × F_0_ + 1 × F_1_ + 2 × F_2_ + 3 × F_3_).

### Micro-CT scanning

After fixed in the formalin, calvarias were scanned using a high-resolution in vivo micro-CT imaging system (ZKKS-MCT-Sharp, Zhongke Kaisheng Medical Technology) and further analysed by the affiliated software (ZZKS-Micro-CT4.1). The radiographic projection was performed under the condition of 60 kV and 667 μA within 240 ms. All the projection frames were recorded five times for average. After the reconstruction 3D images were obtained by the bundled manufacturer’s reconstruction software, a 1 × 3 × 3 mm^3^ region was set as region of interest (ROI) for gauging bone mineral density (BMD), bone volume (BV)/total volume (TV) and osteolysis area.

### Statistics

Data were analysed by GraphPad Prism (version 8.0.2) and displayed in the form of mean ± SEM. All values were assessed by the Kolmogorov–Smirnov test to verify data normality. T-test was performed to compare the statistical difference between two groups while One-way ANOVA or two-way ANOVA was performed for more than two groups. The statistical data were compared and *p* value < 0.05 was considered statistically significant compared with the negative group. *p < 0.05, **p < 0.01, ***p < 0.001. For synovium staining, seven patients with prosthetic aseptic loosening and eight patients with femoral head necrosis were included to ensure the reliability. For vivo study, six mice were included to ensure the reliability. For vitro experiments, assays repeated three time to ensure the reliability.

## Supplementary information

Supplementary materials- merged western blots

Fig. 2C-GAPDH for p110δ

Fig. 2C-p110δ

Fig. 3C-IL-6

Fig. 3C-TNFα

Fig. 3C-β tubulin for IL-6

Fig. 3C-β tubulin for TNFα

Fig.3H-GAPDH for MAPK signaling pathway

Fig.3H-p-Erk

Fig.3H-p-JNK

Fig.3I-p-IκBα

Fig.3H-p-p38

Fig.3I-p-p65

Fig.3I-β Tubulin for NF-κB signaling pathway

Fig.4C-GAPDH for p110δ

Fig.4C-p110δ

Fig.5D-IκBα

Fig.5D-p-IκBα

Fig.5D-p-p65

Fig.5D-p65

Fig.5D-β Tubulin for NF-κB signaling pathway

Fig.6D-GAPDH for KLF4

Fig.6D-KLF4

Fig.6H-GAPDH for p110δ

Fig.6H-p110δ

Fig.6I-IκBα

Fig.6I-p-IκBα

Fig.6I-p-p65

Fig.6I-p65

Fig.6I-β Tubulin for NF-κB signaling pathway

Supplemental material of figures and figures caption that are not shown in the manuscript

## Data Availability

The data relative to the findings of this study are available from corresponding author or the Supplementary Materials section.
