# Supplementary figures and images for: MiR-92a/KLF4/p110δ regulates titanium particles-induced macrophages inflammation and osteolysis
Source: Cell Death Discov. 2022 Apr 13;8:197. doi: 10.1038/s41420-022-00999-2 (PMC9007998; doi:10.1038/s41420-022-00999-2)

Original Western Blots

Fig. 2C

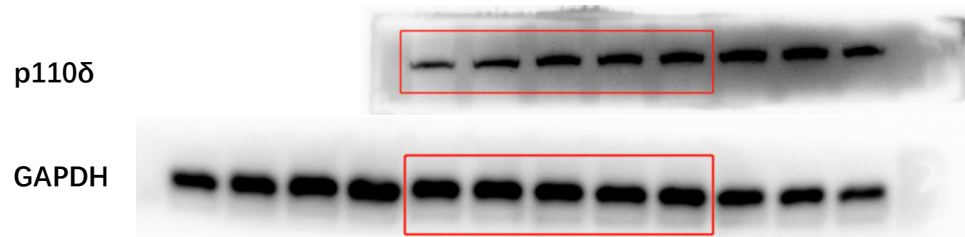

Fig. 3C

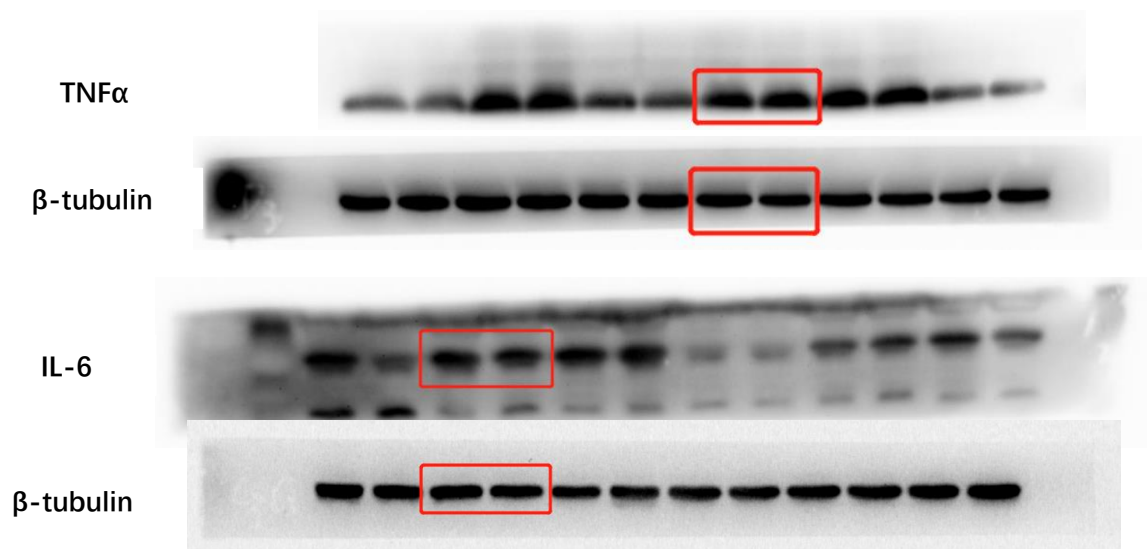

Fig. 3H

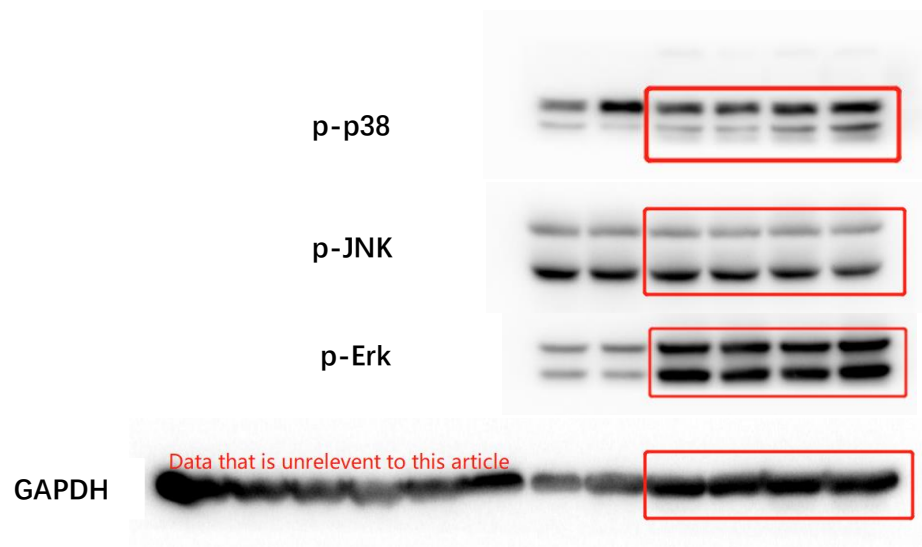

Fig. 3I

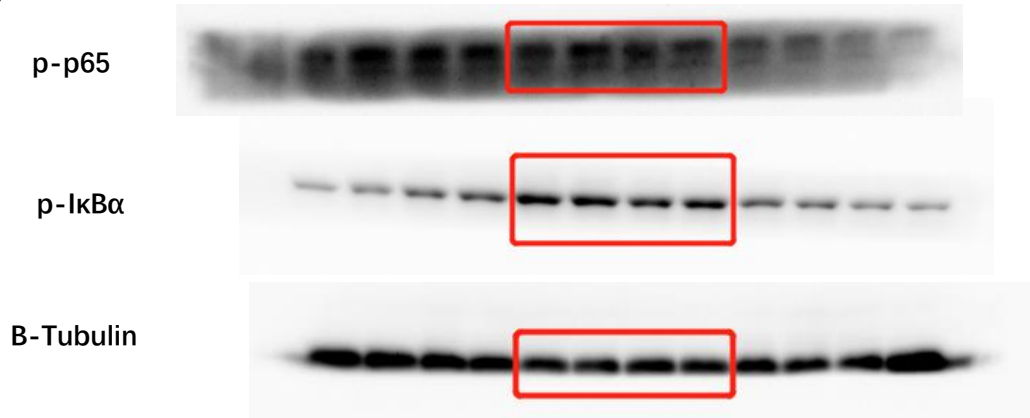

Fig. 4C

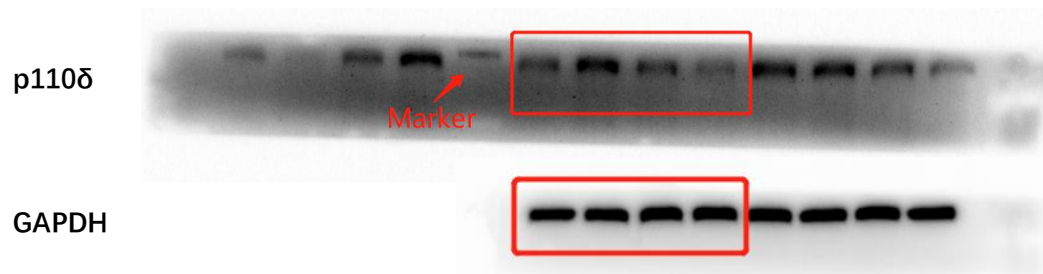

Fig. 5D

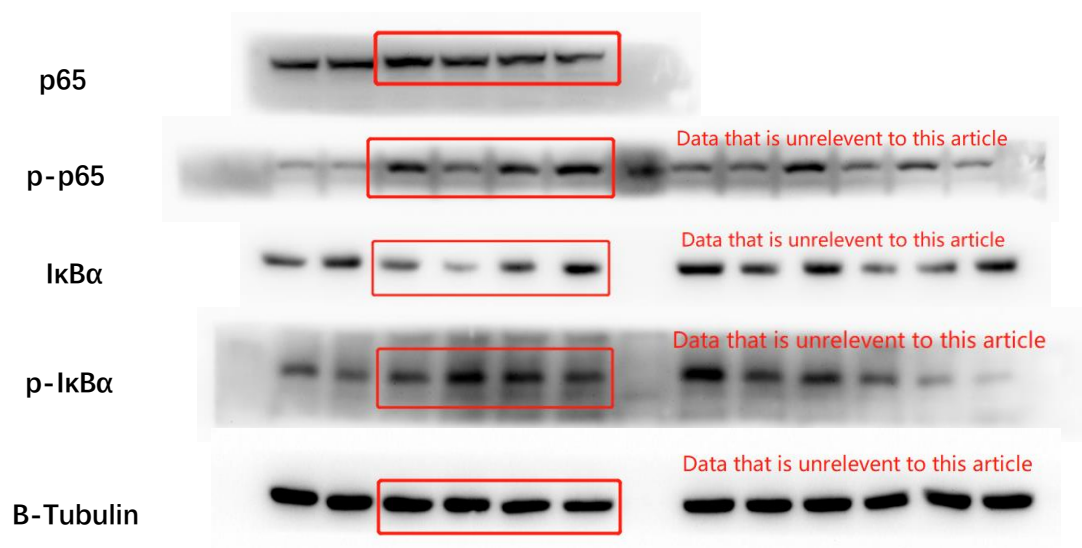

Fig. 6D

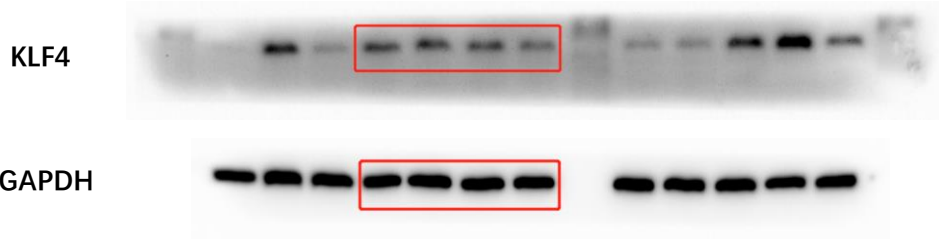

Fig. 6H

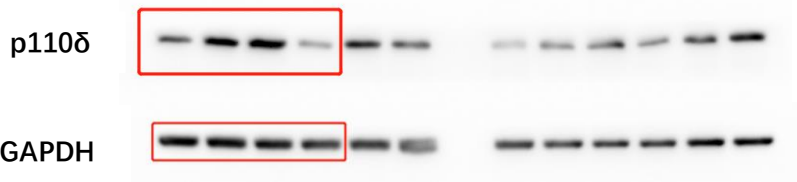

Fig. 6I

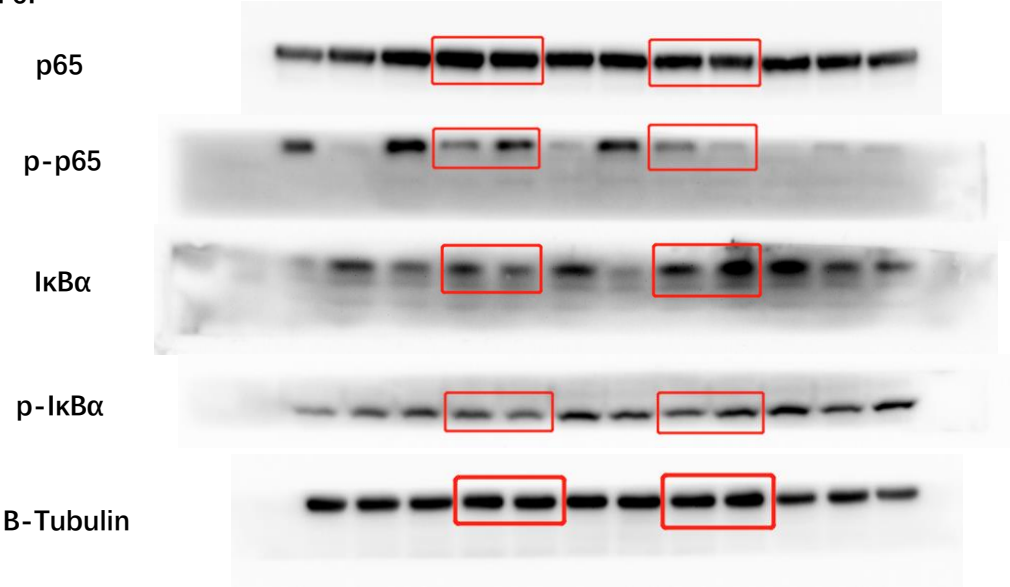

Fig. S2H

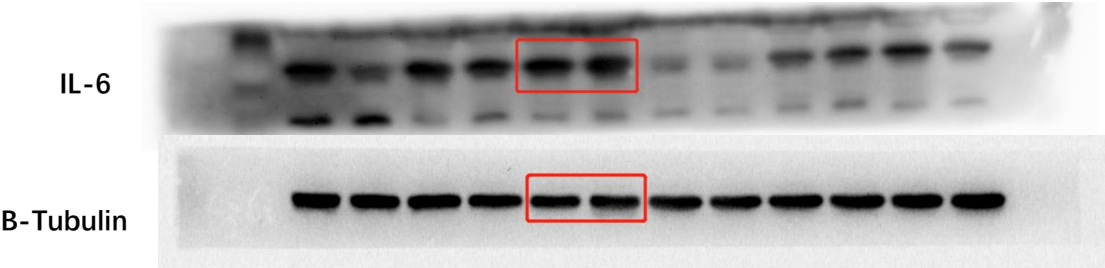

TNF $\alpha$

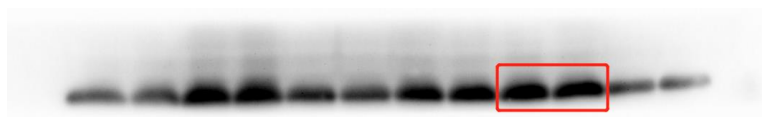

B-Tubulin

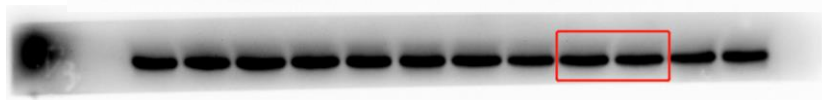

Supplement: Supplementary file 1 — Supplementary materials- merged western blots [file 41420_2022_999_MOESM1_ESM.pdf]

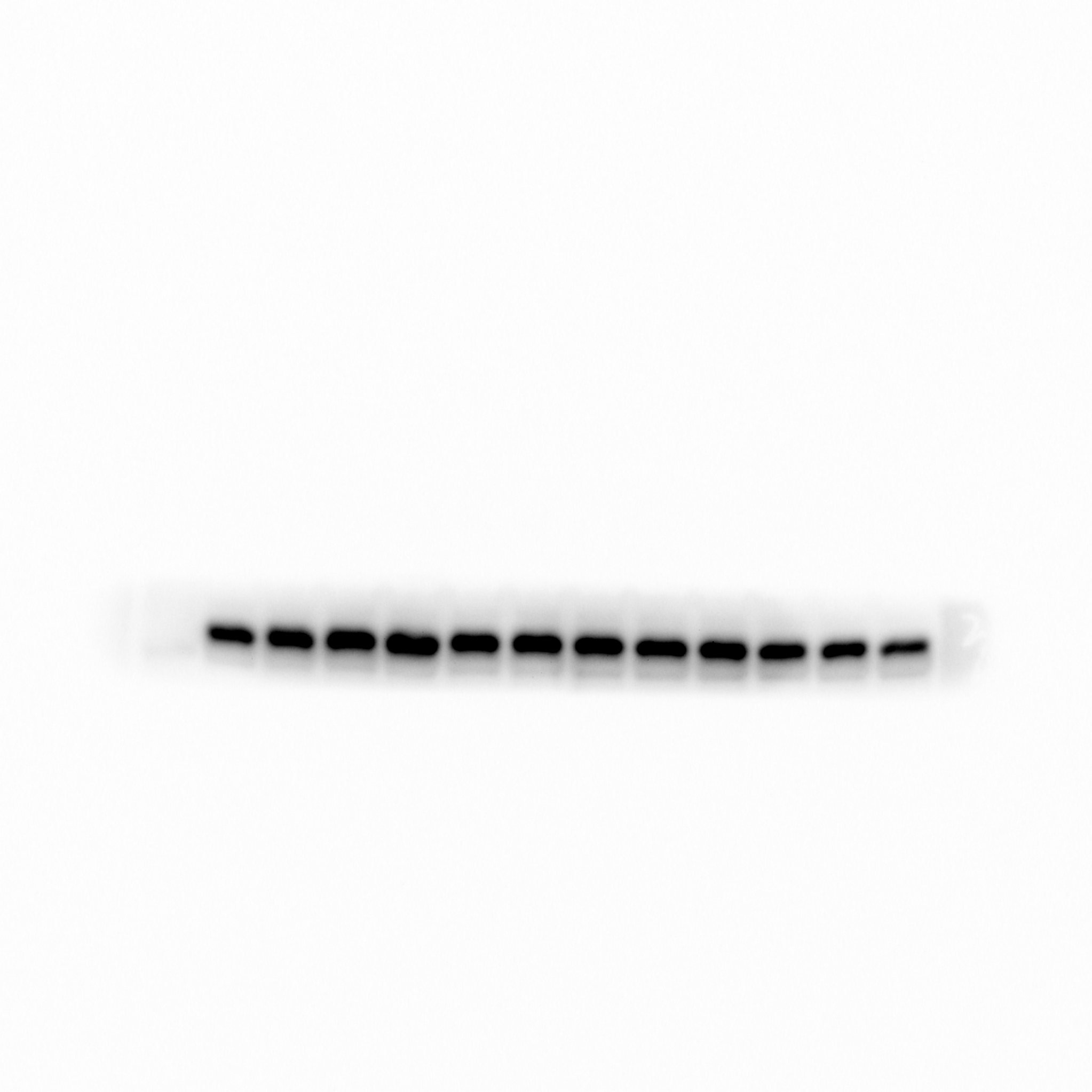

Supplement: Supplementary file 2 — Fig. 2C-GAPDH for p110δ [file 41420_2022_999_MOESM2_ESM.tif]

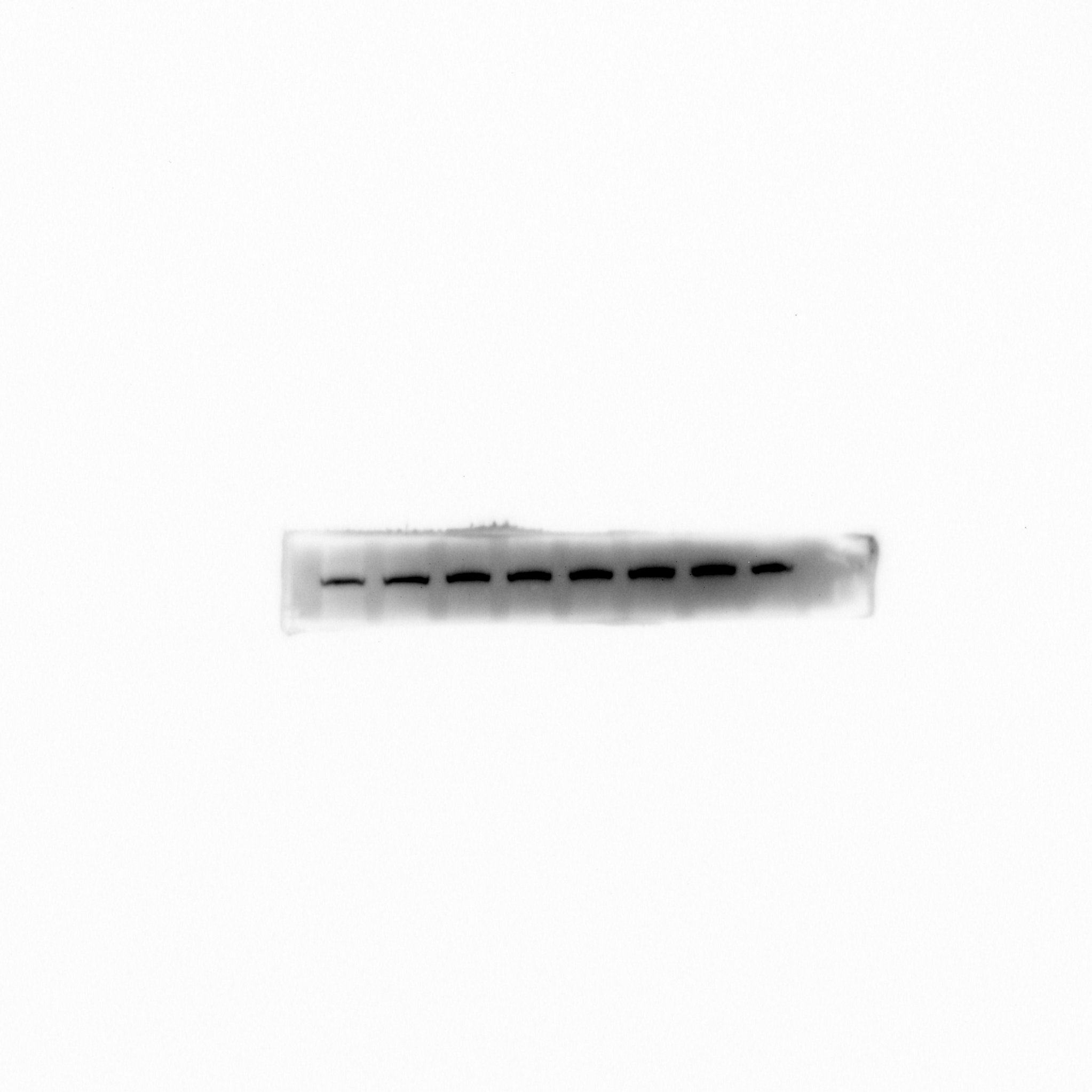

Supplement: Supplementary file 3 — Fig. 2C-p110δ [file 41420_2022_999_MOESM3_ESM.tif]

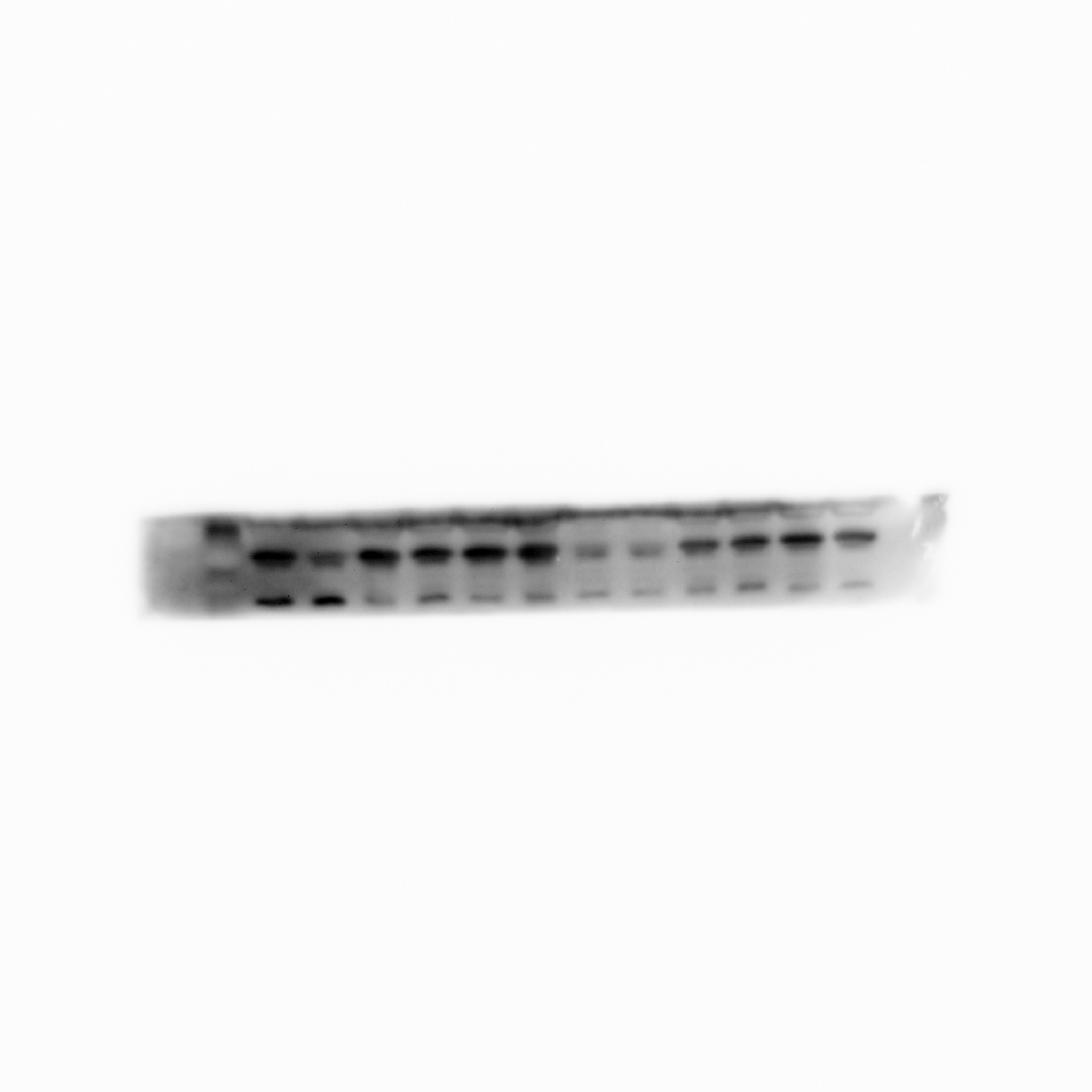

Supplement: Supplementary file 4 — Fig. 3C-IL-6 [file 41420_2022_999_MOESM4_ESM.tif]

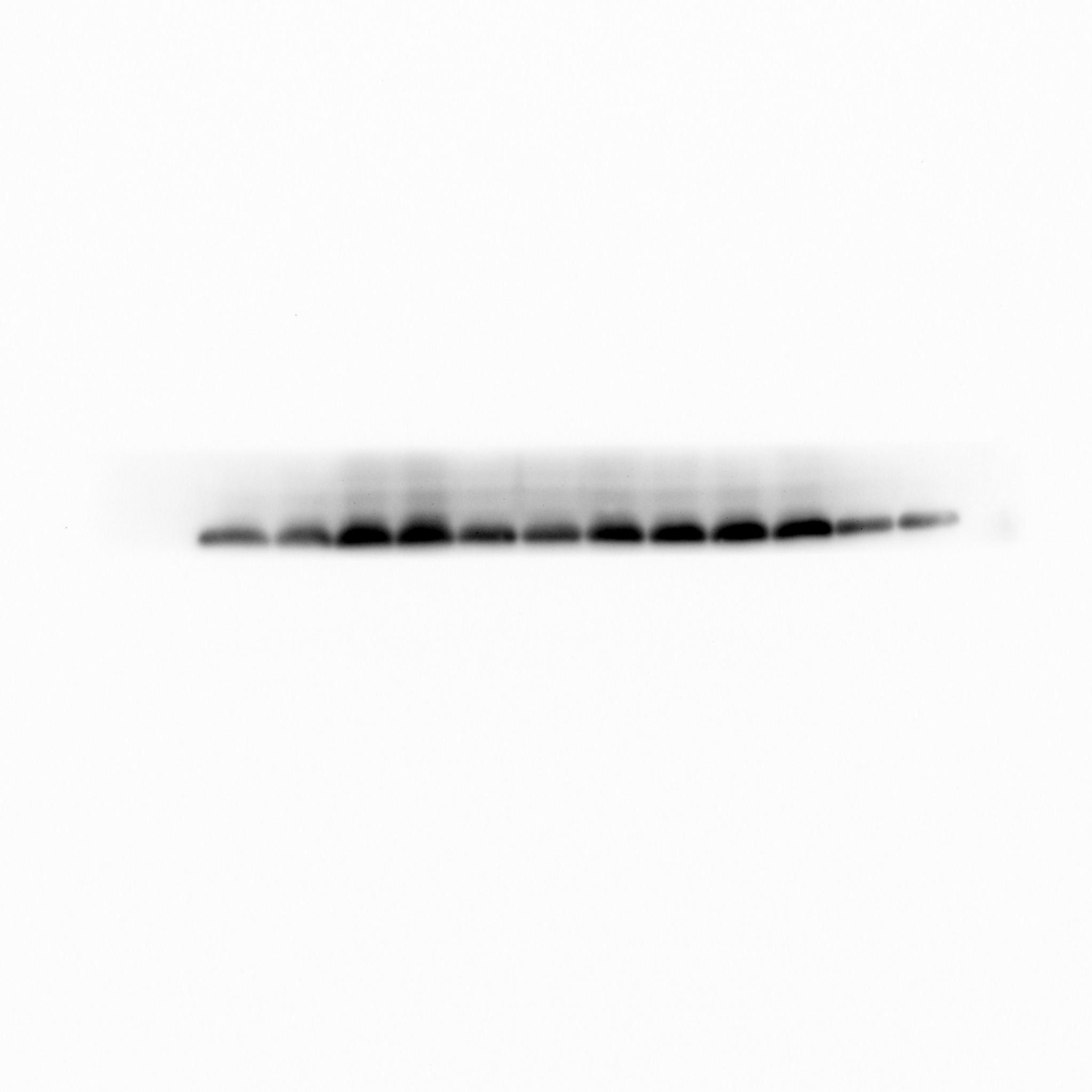

Supplement: Supplementary file 5 — Fig. 3C-TNFα [file 41420_2022_999_MOESM5_ESM.tif]

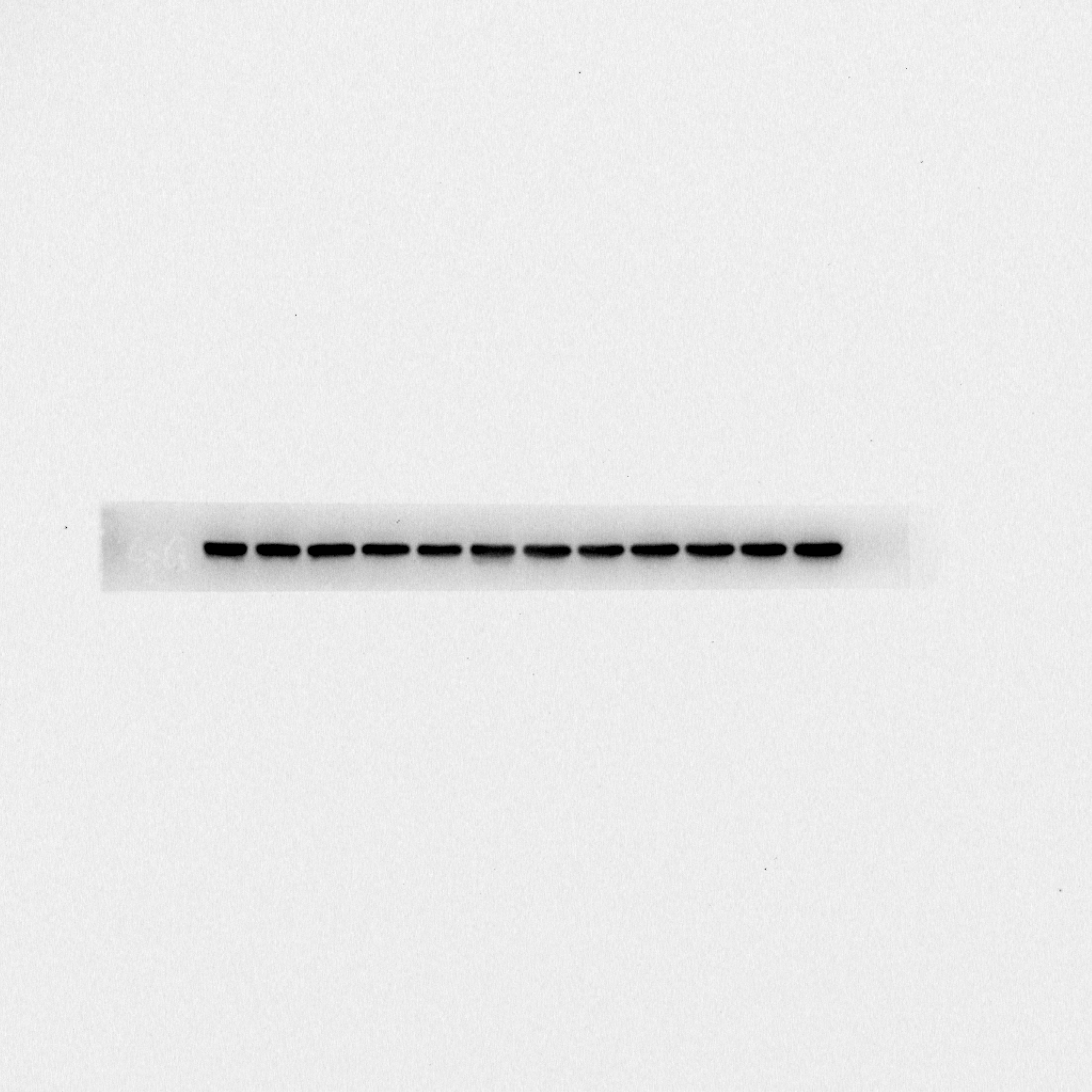

Supplement: Supplementary file 6 — Fig. 3C-β tubulin for IL-6 [file 41420_2022_999_MOESM6_ESM.tif]

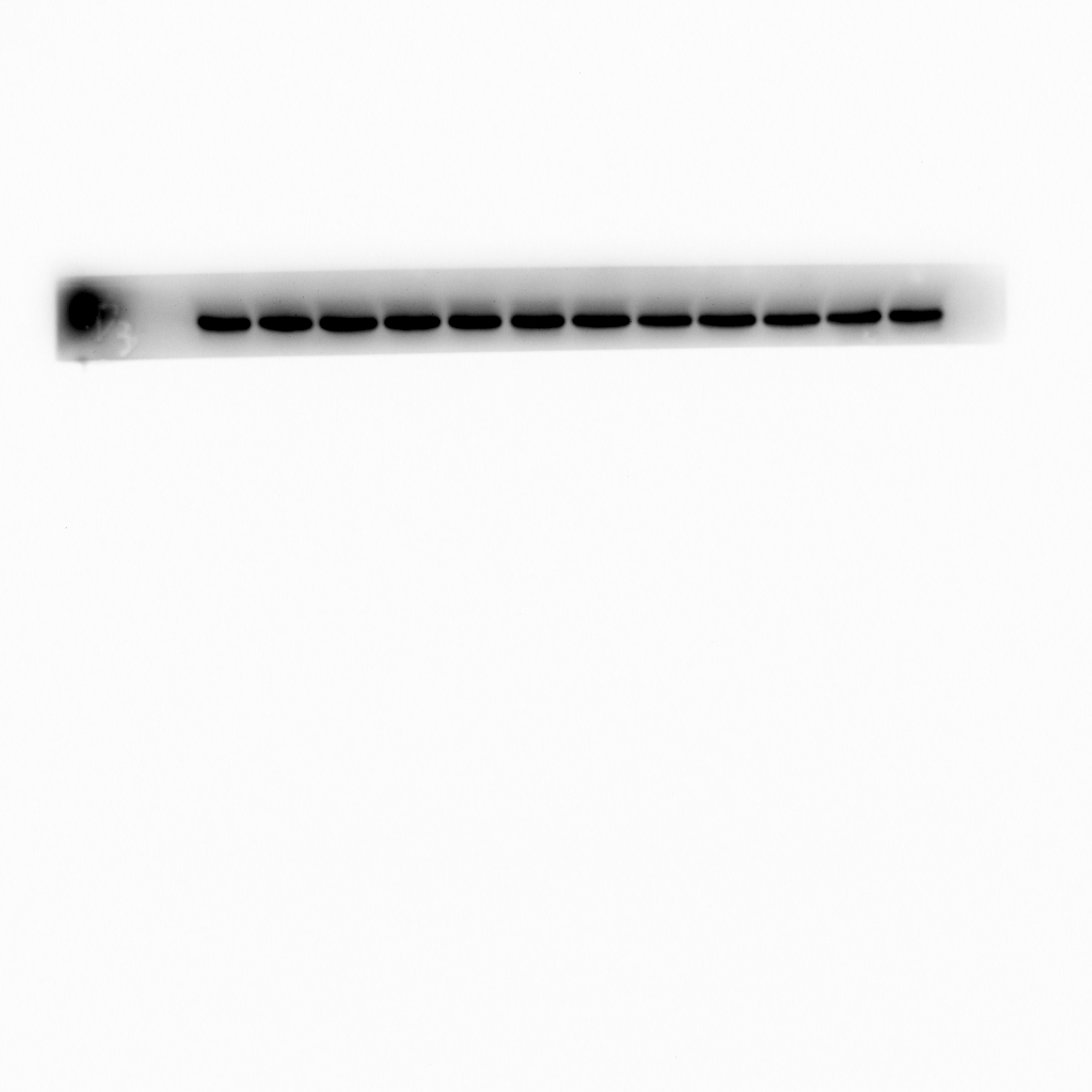

Supplement: Supplementary file 7 — Fig. 3C-β tubulin for TNFα [file 41420_2022_999_MOESM7_ESM.tif]

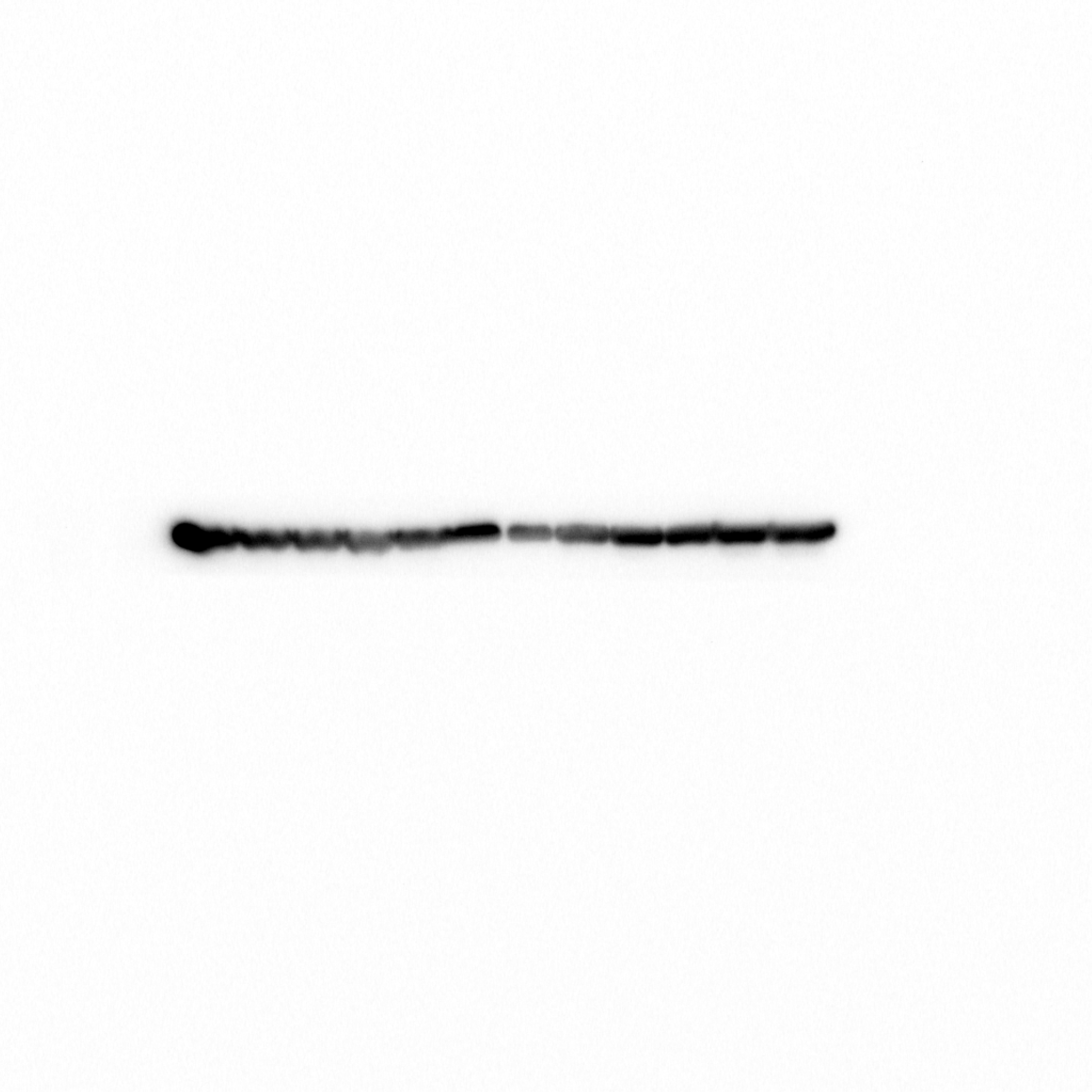

Supplement: Supplementary file 8 — Fig.3H-GAPDH for MAPK signaling pathway [file 41420_2022_999_MOESM8_ESM.tif]

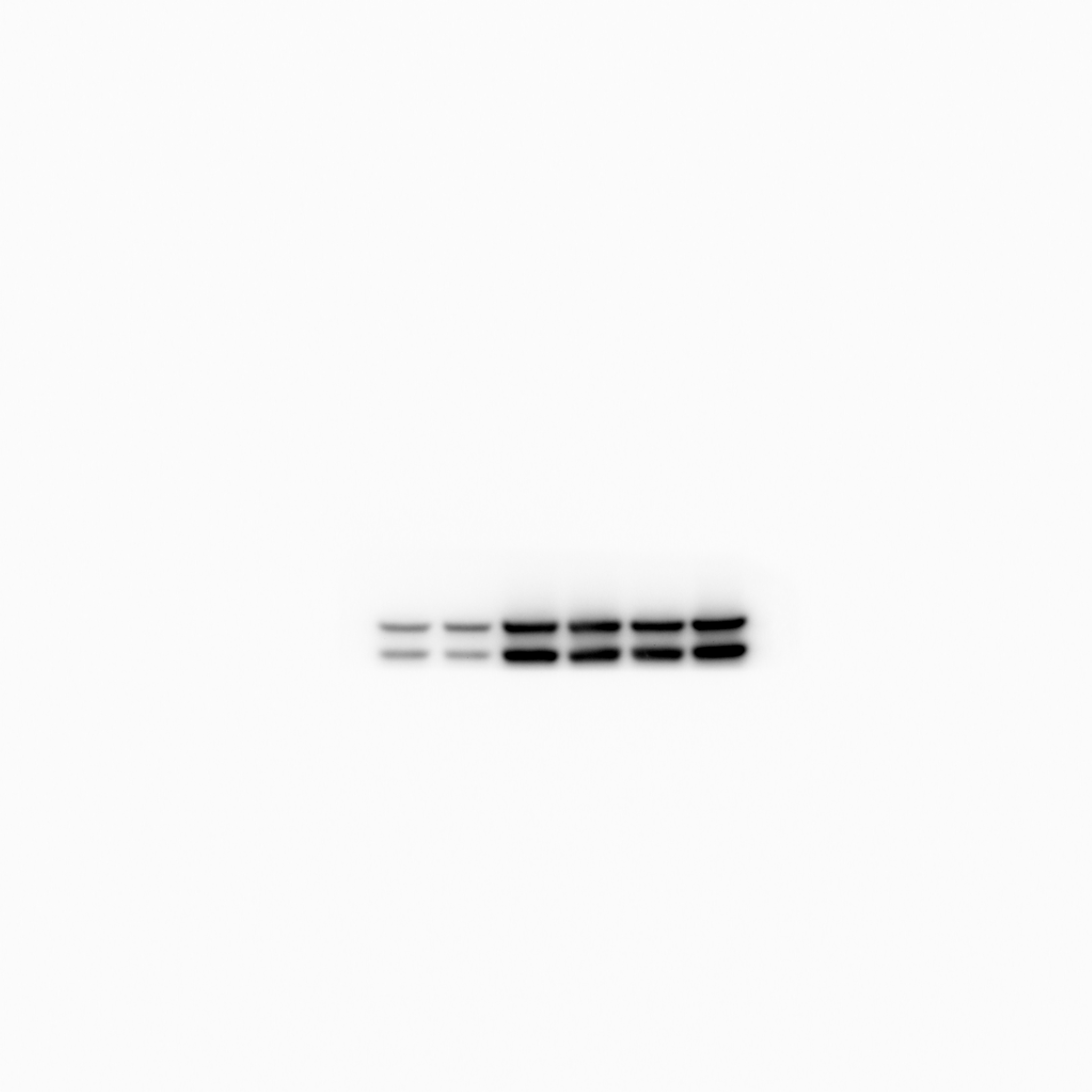

Supplement: Supplementary file 9 — Fig.3H-p-Erk [file 41420_2022_999_MOESM9_ESM.tif]

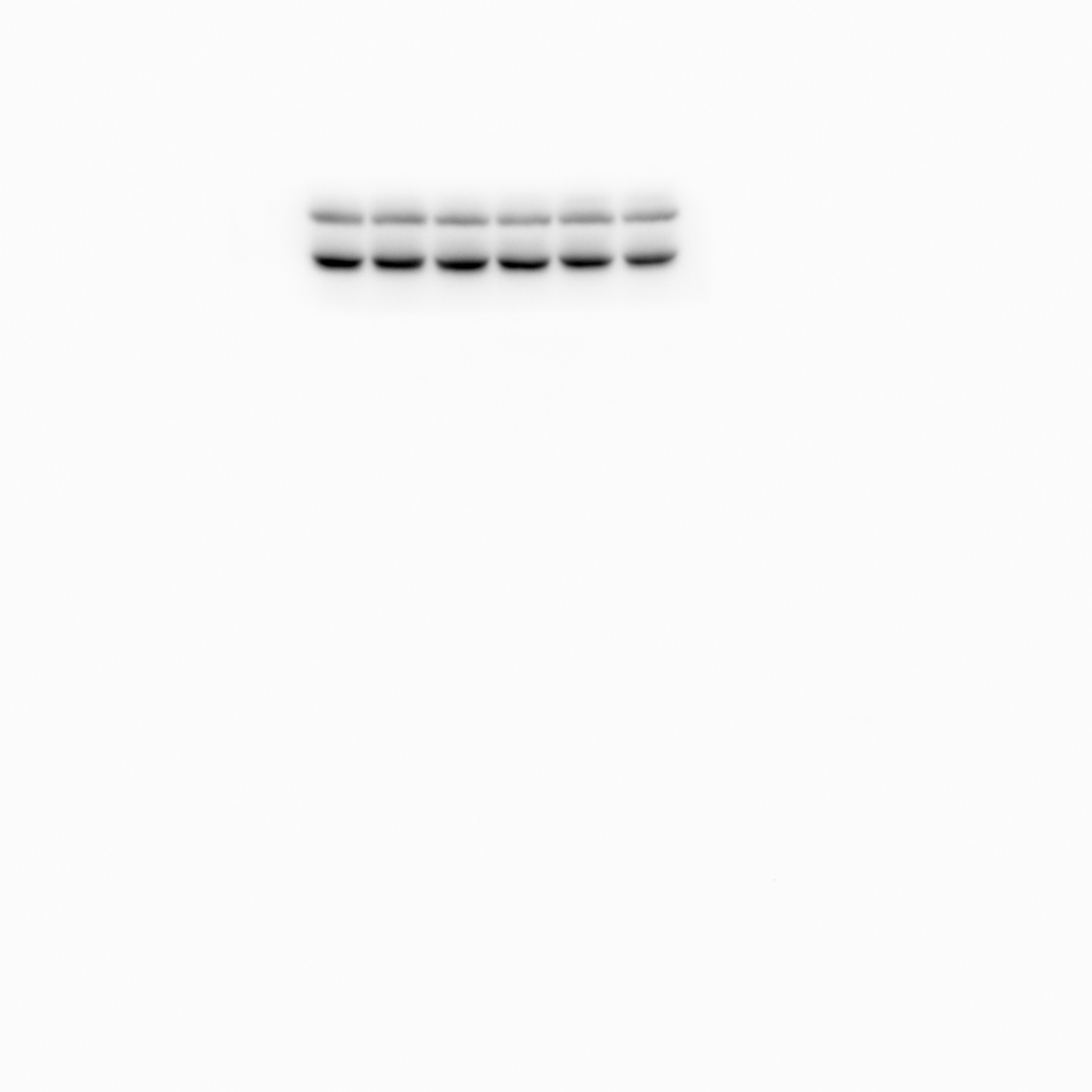

Supplement: Supplementary file 10 — Fig.3H-p-JNK [file 41420_2022_999_MOESM10_ESM.tif]

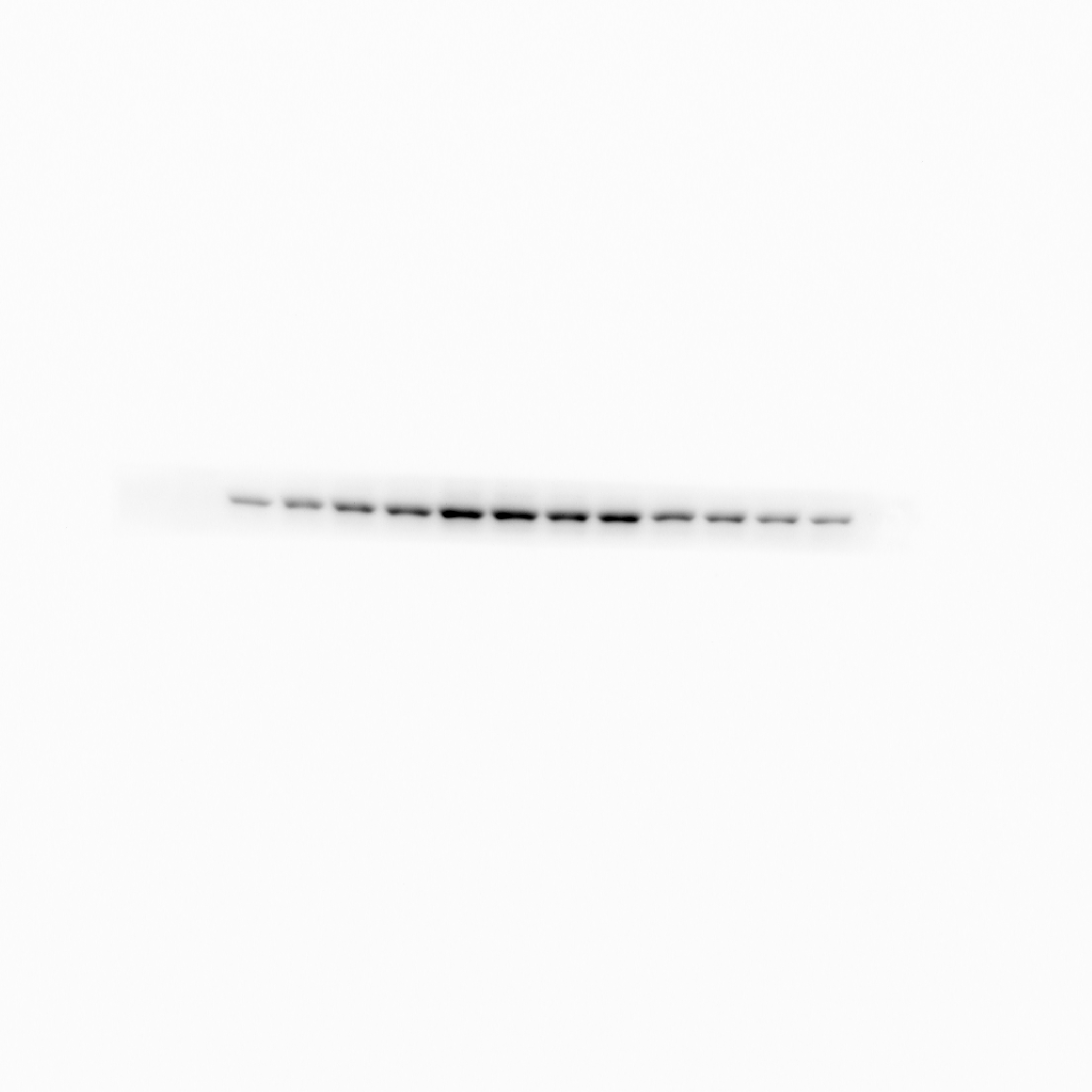

Supplement: Supplementary file 11 — Fig.3I-p-IκBα [file 41420_2022_999_MOESM11_ESM.tif]

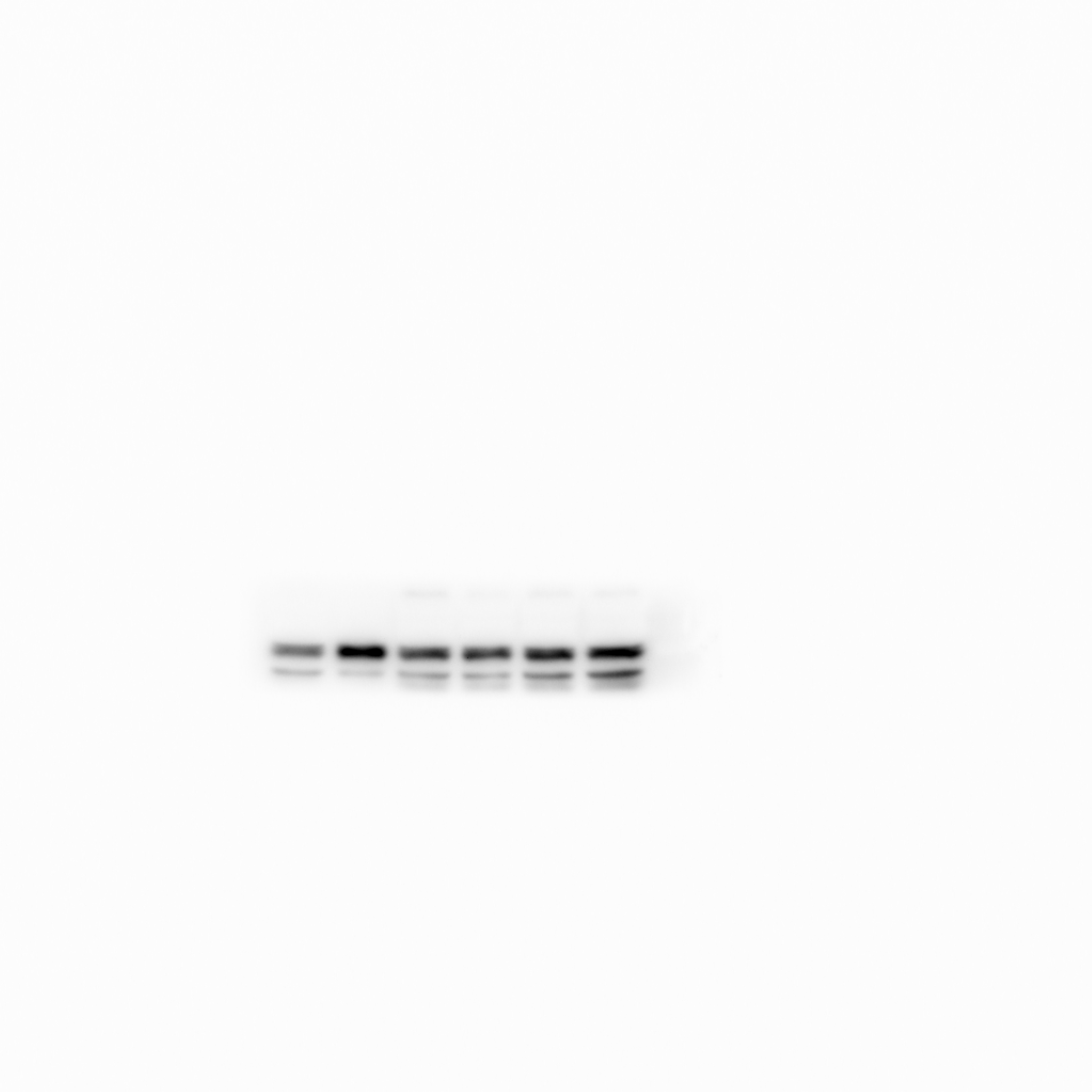

Supplement: Supplementary file 12 — Fig.3H-p-p38 [file 41420_2022_999_MOESM12_ESM.tif]

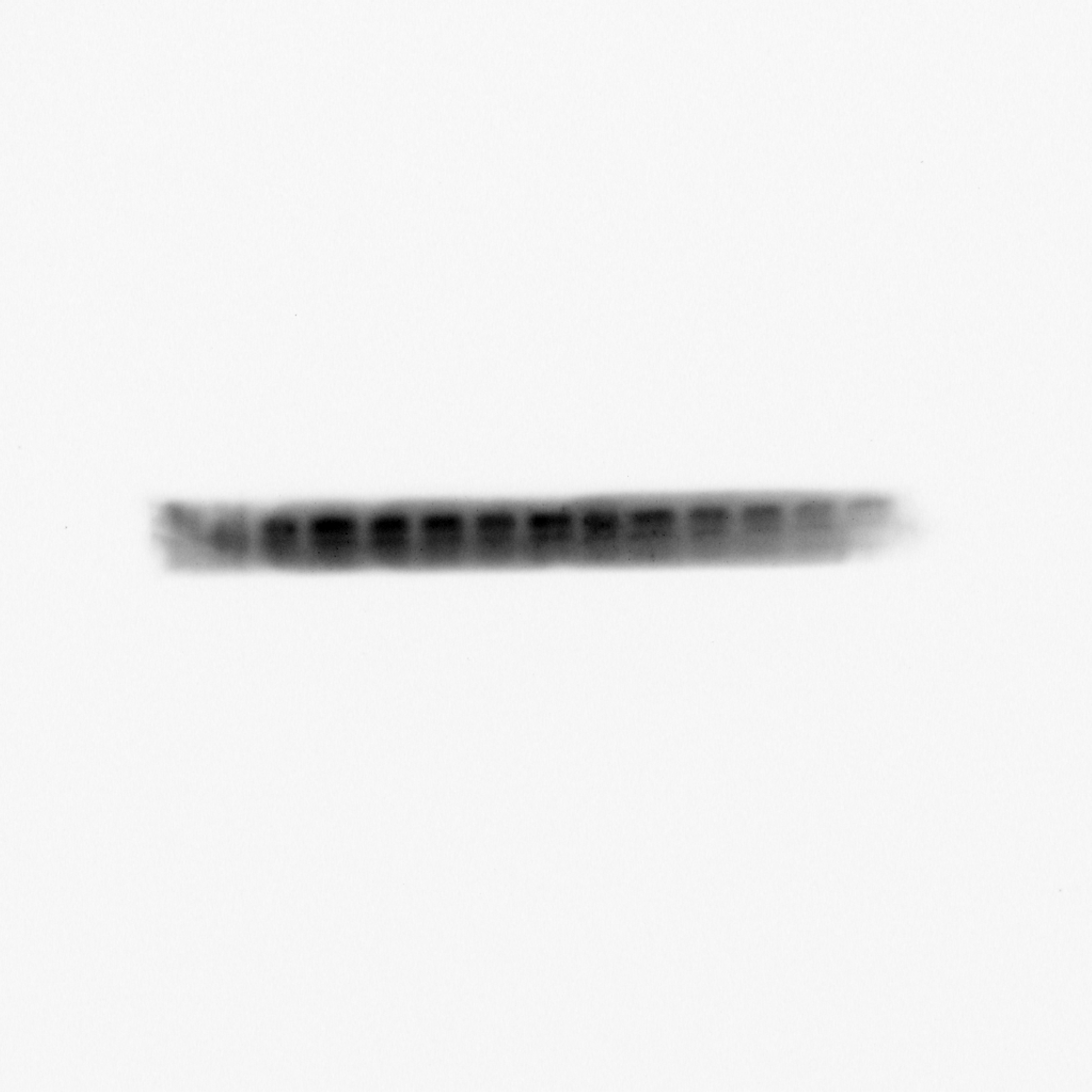

Supplement: Supplementary file 13 — Fig.3I-p-p65 [file 41420_2022_999_MOESM13_ESM.tif]

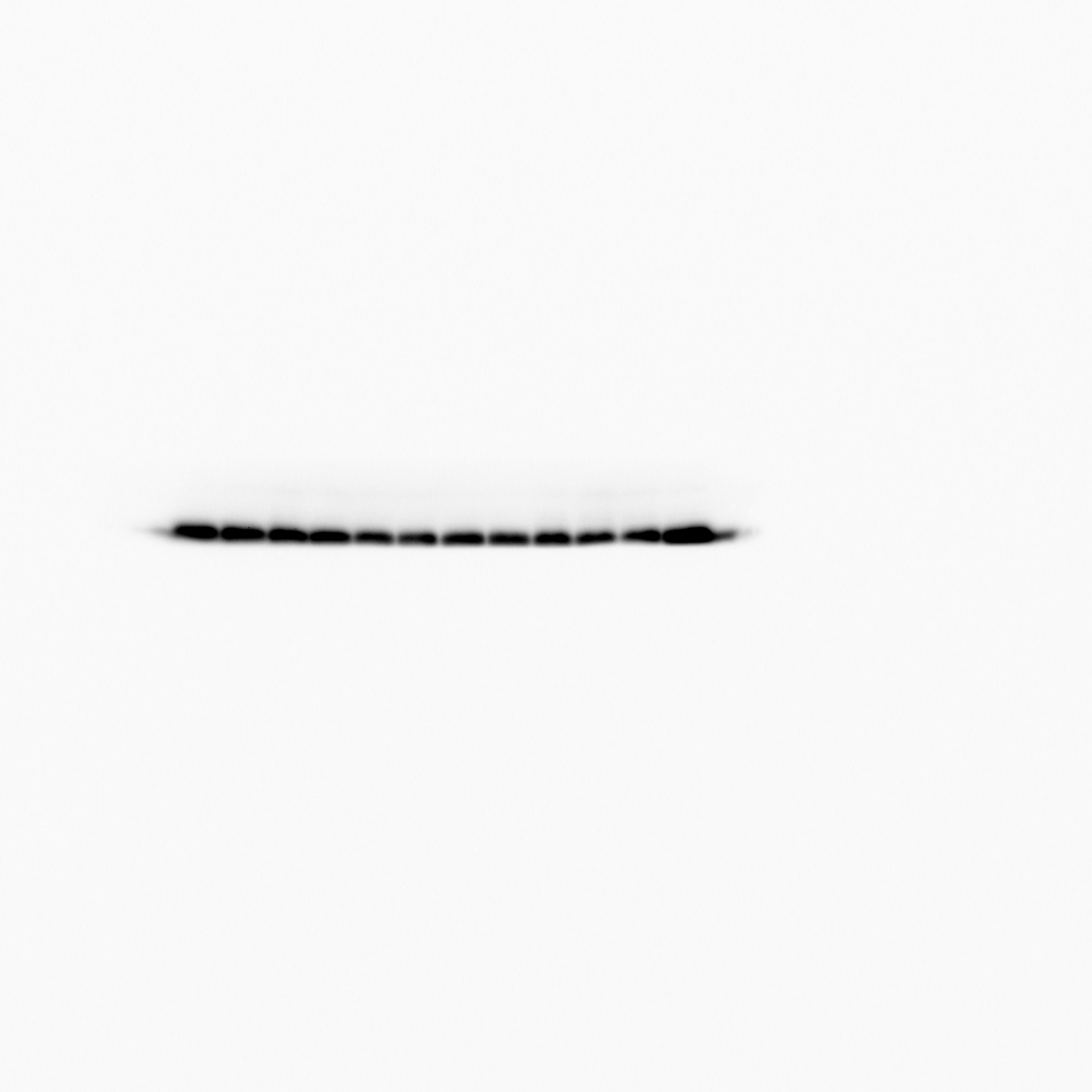

Supplement: Supplementary file 14 — Fig.3I-β Tubulin for NF-κB signaling pathway [file 41420_2022_999_MOESM14_ESM.tif]

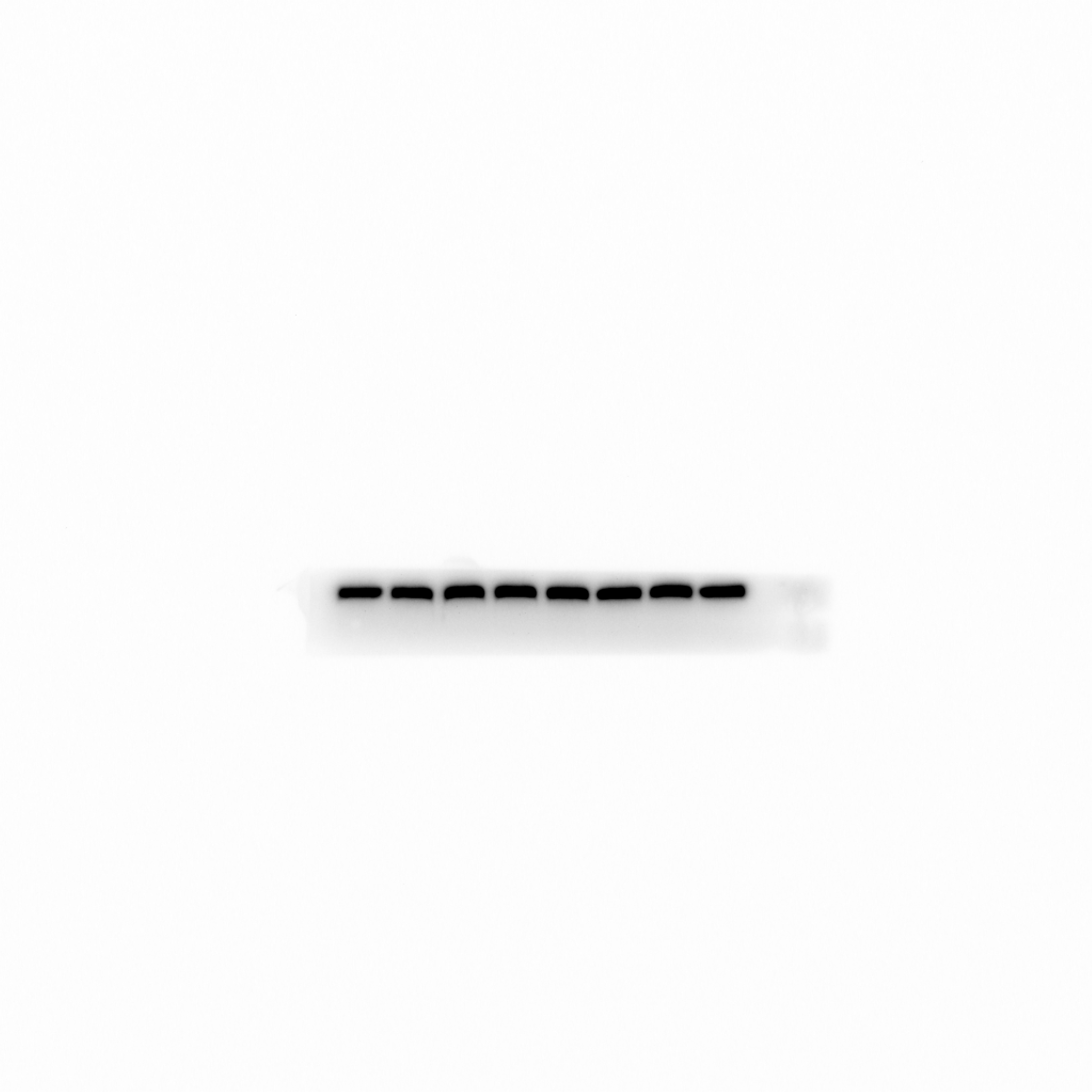

Supplement: Supplementary file 15 — Fig.4C-GAPDH for p110δ [file 41420_2022_999_MOESM15_ESM.tif]

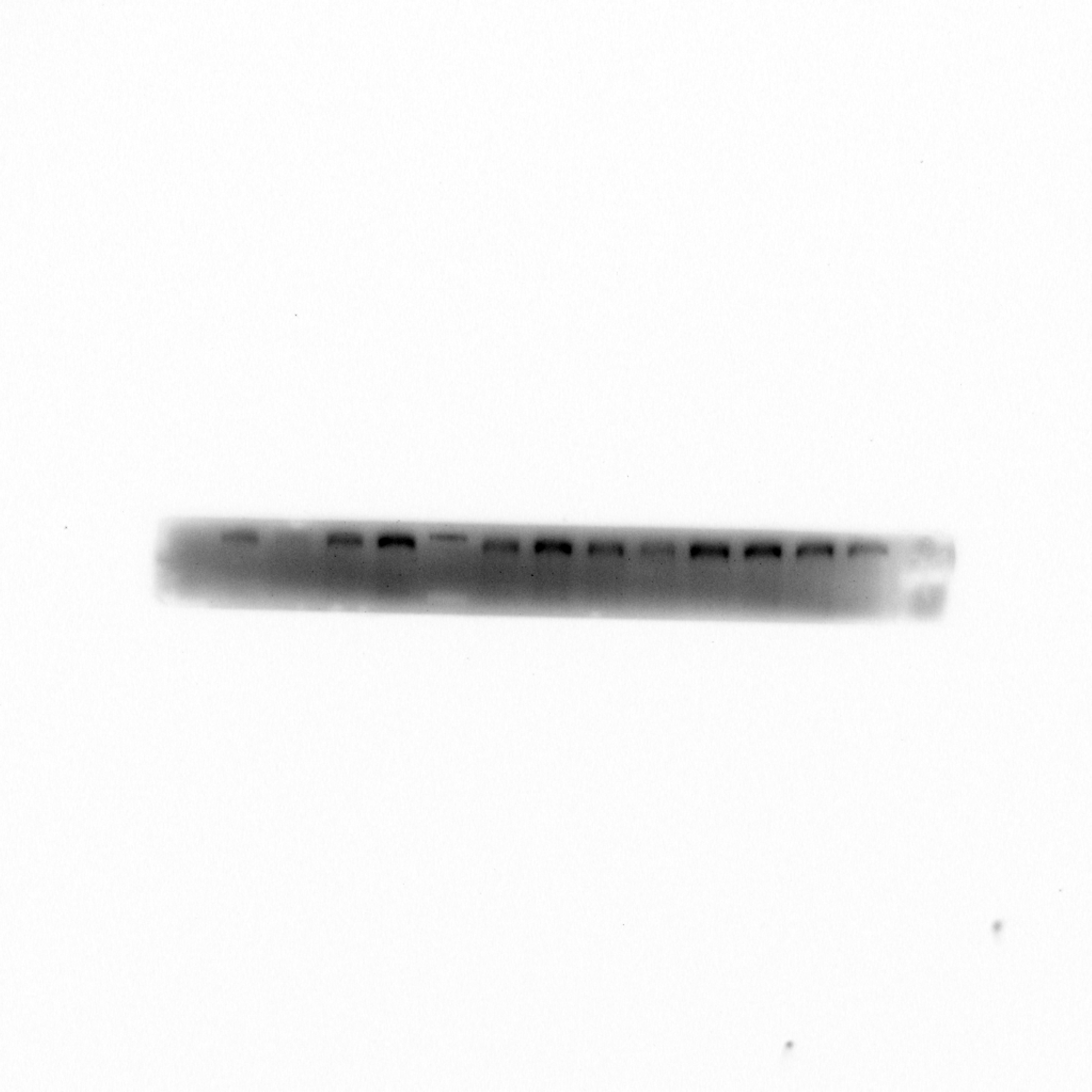

Supplement: Supplementary file 16 — Fig.4C-p110δ [file 41420_2022_999_MOESM16_ESM.tif]

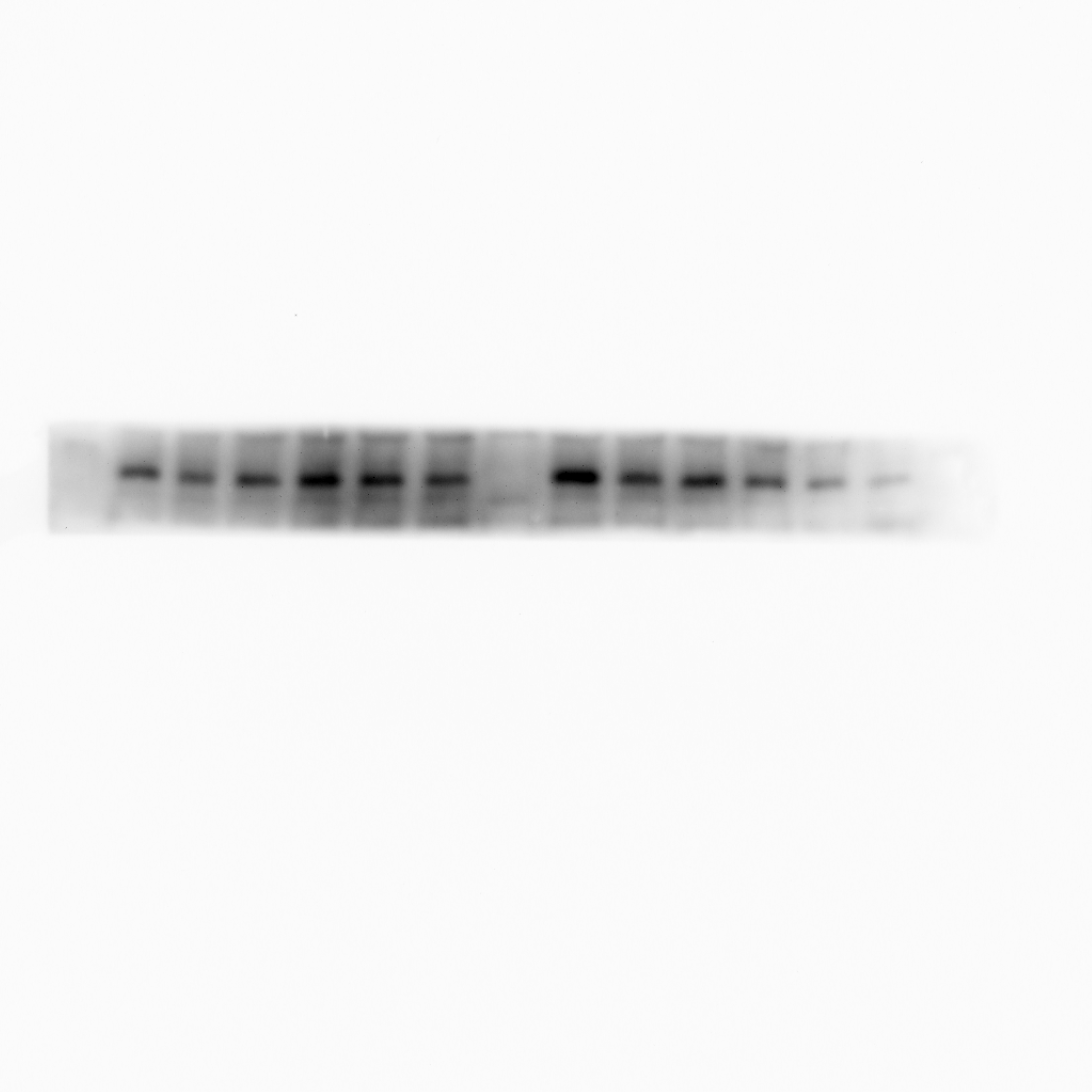

Supplement: Supplementary file 17 — Fig.5D-IκBα [file 41420_2022_999_MOESM17_ESM.tif]

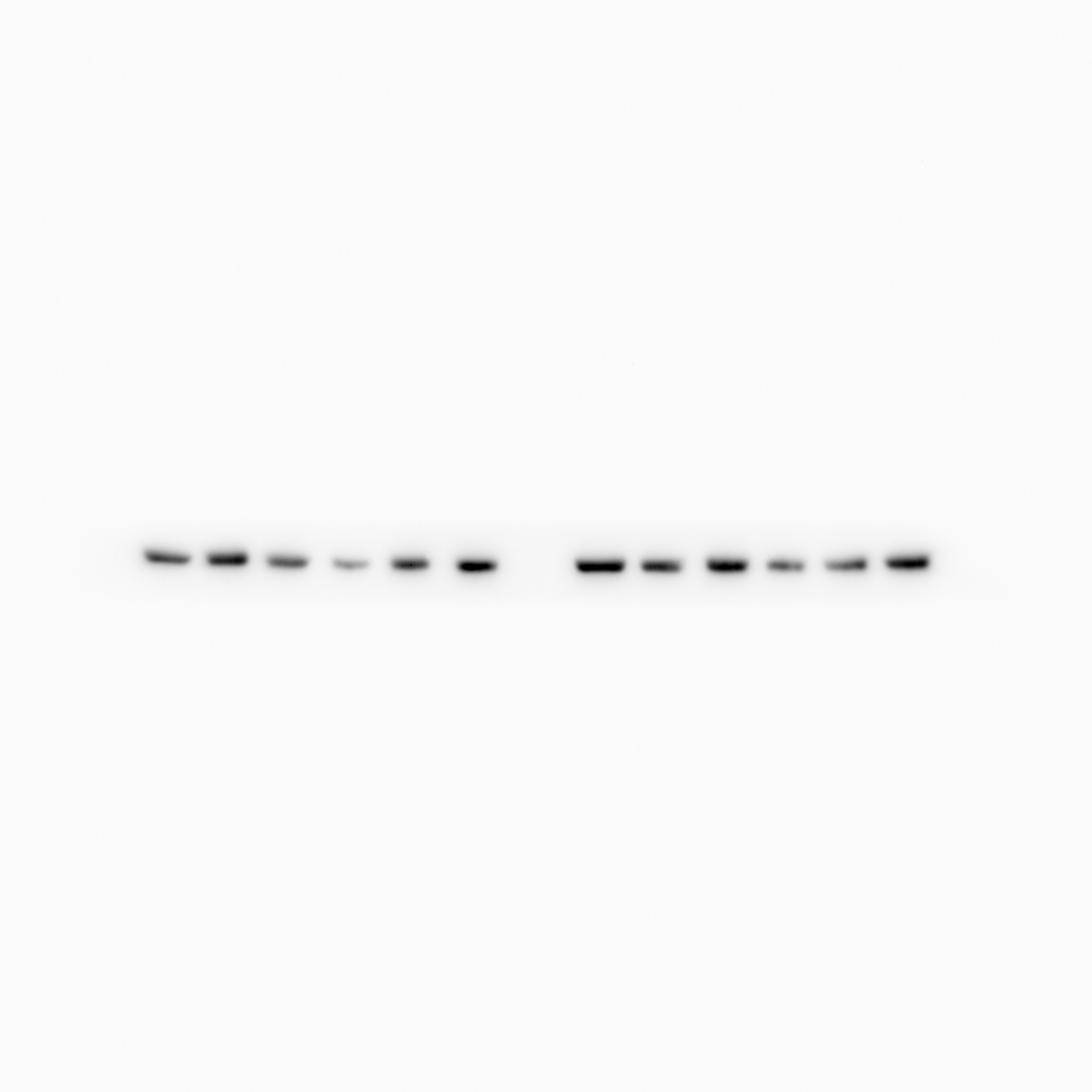

Supplement: Supplementary file 18 — Fig.5D-p-IκBα [file 41420_2022_999_MOESM18_ESM.tif]

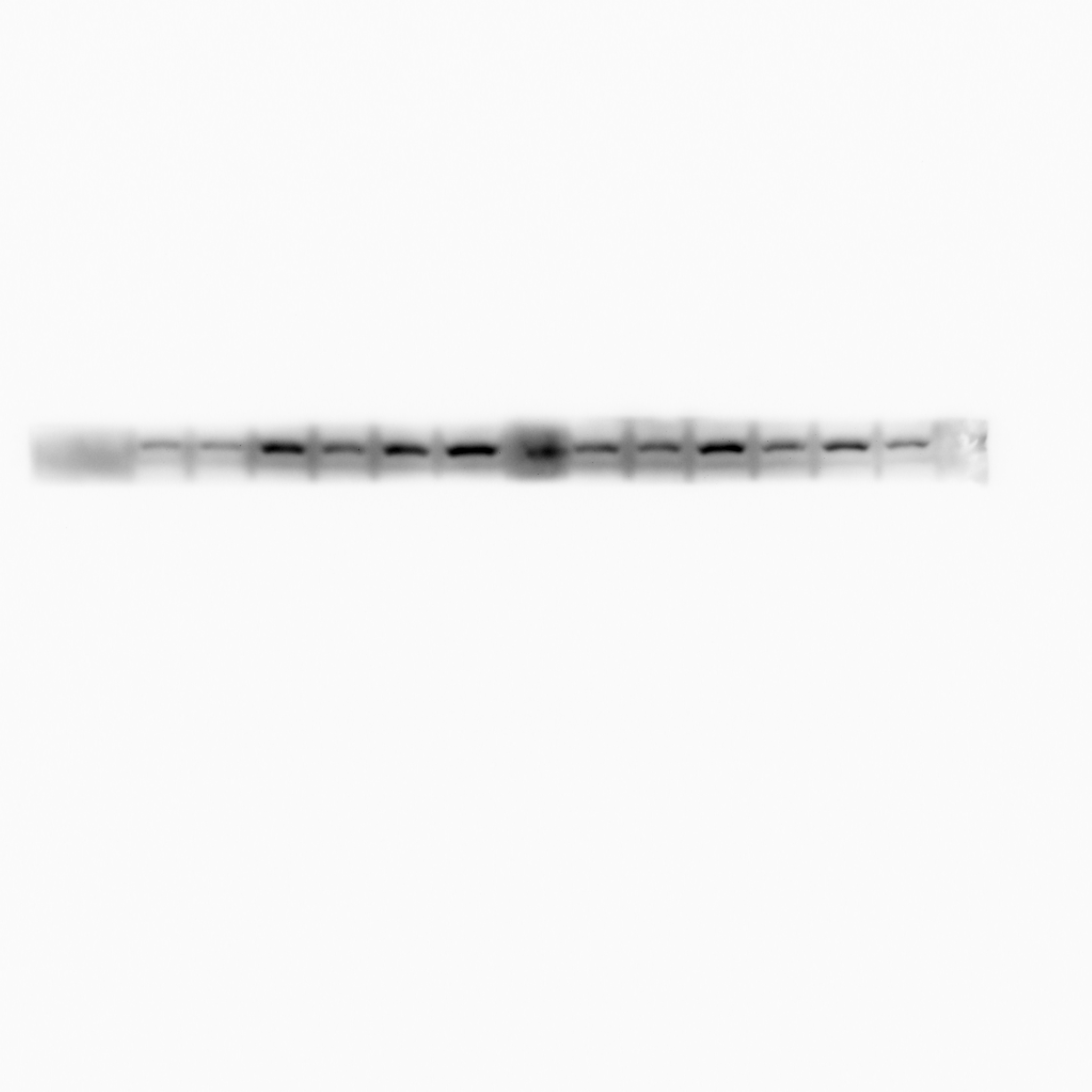

Supplement: Supplementary file 19 — Fig.5D-p-p65 [file 41420_2022_999_MOESM19_ESM.tif]

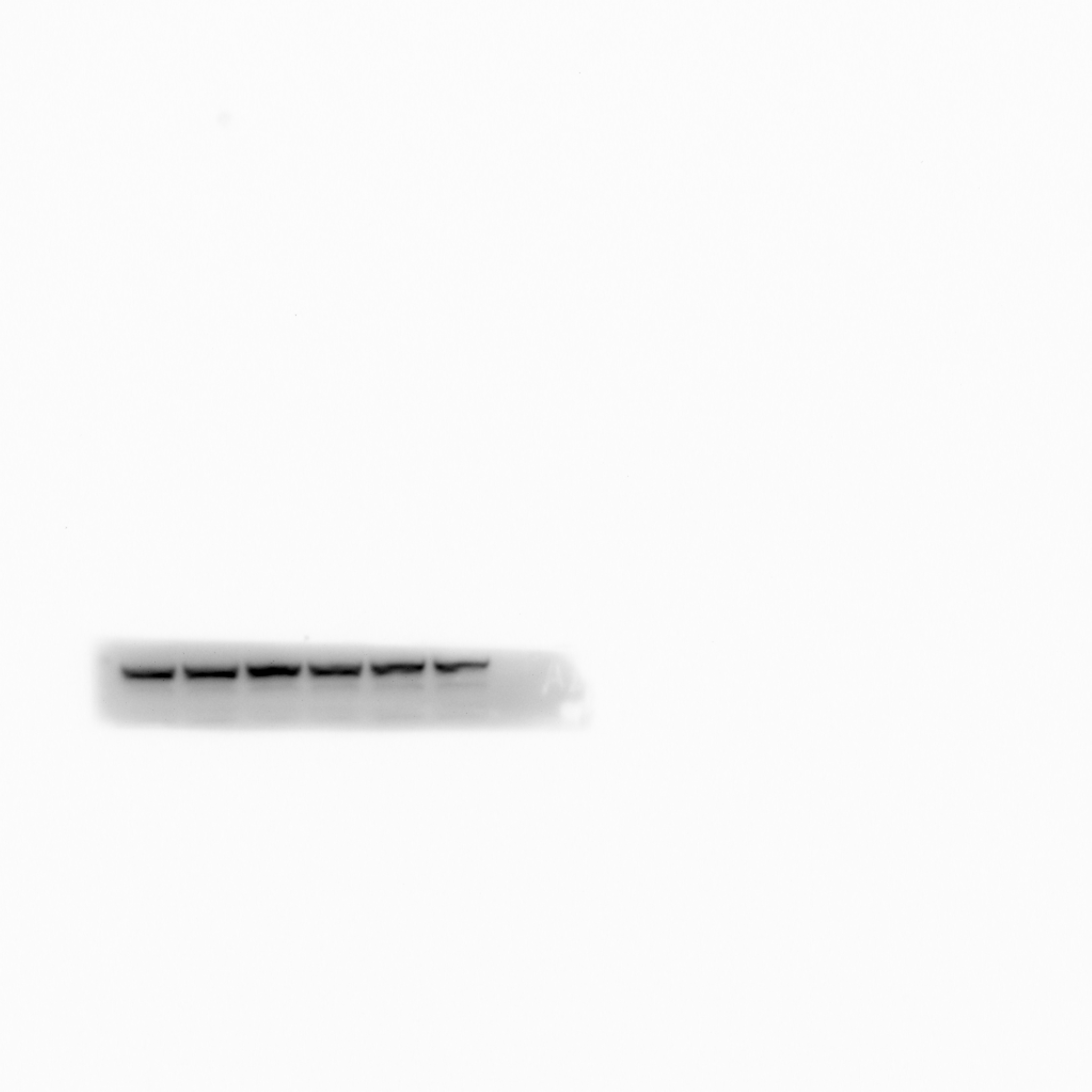

Supplement: Supplementary file 20 — Fig.5D-p65 [file 41420_2022_999_MOESM20_ESM.tif]

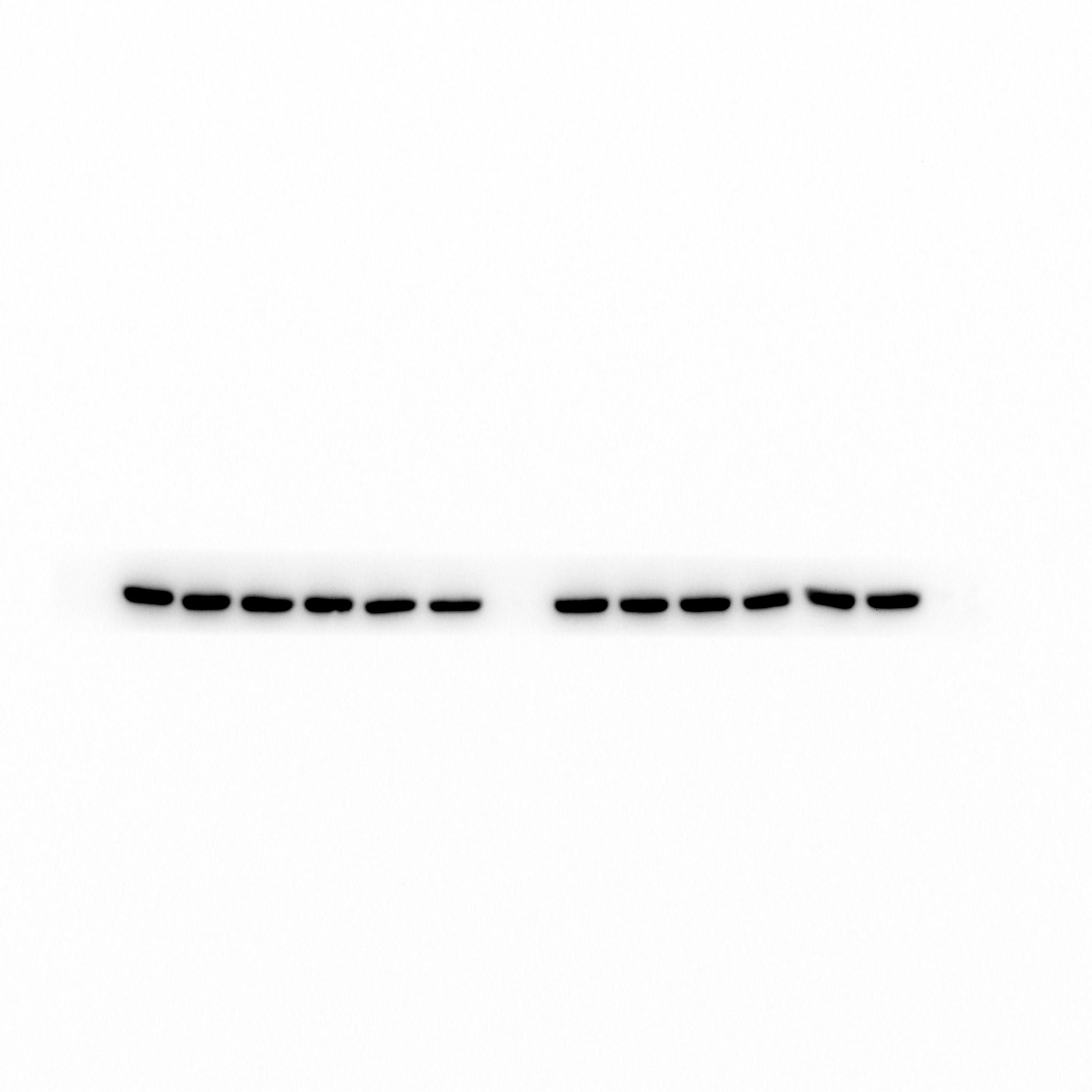

Supplement: Supplementary file 21 — Fig.5D-β Tubulin for NF-κB signaling pathway [file 41420_2022_999_MOESM21_ESM.tif]

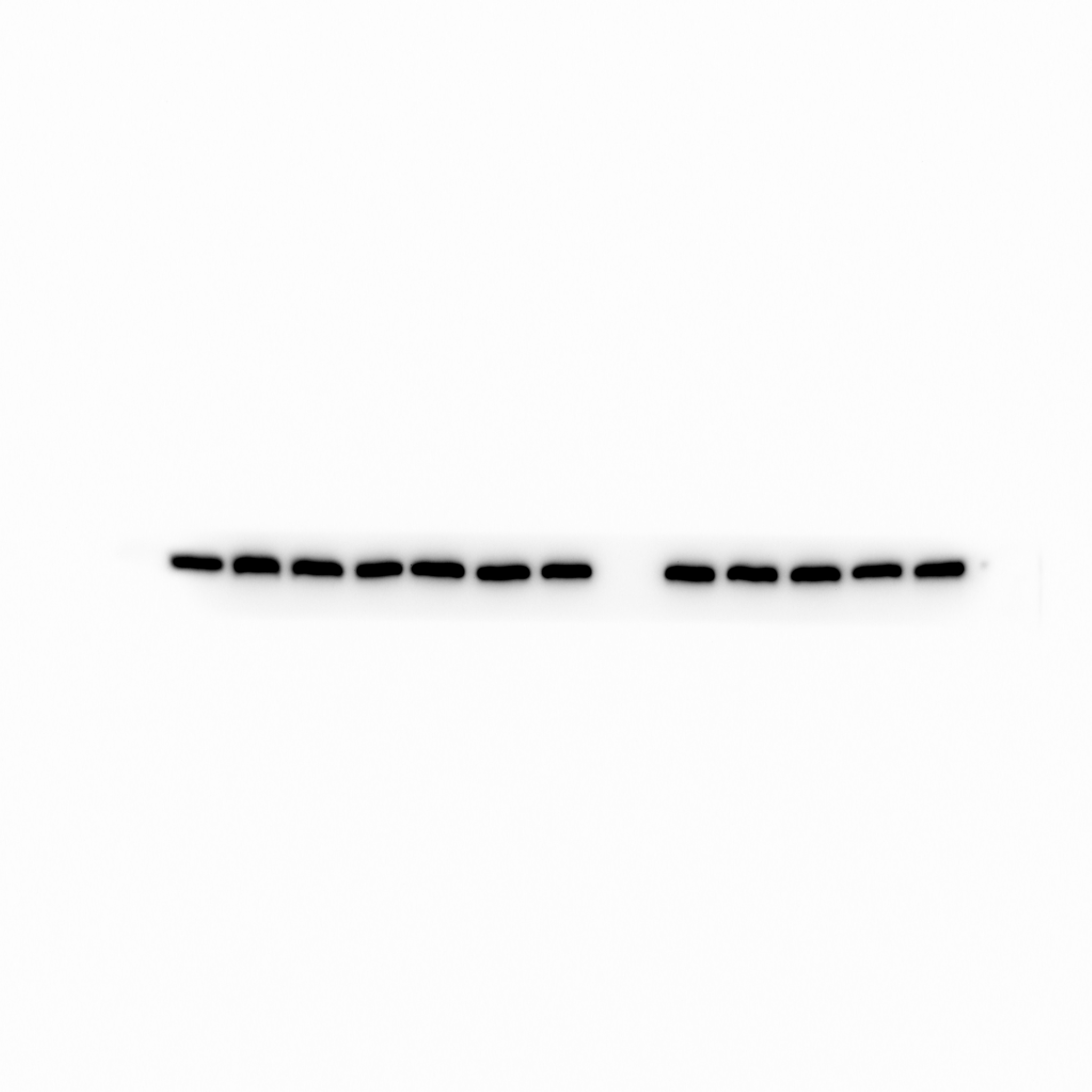

Supplement: Supplementary file 22 — Fig.6D-GAPDH for KLF4 [file 41420_2022_999_MOESM22_ESM.tif]

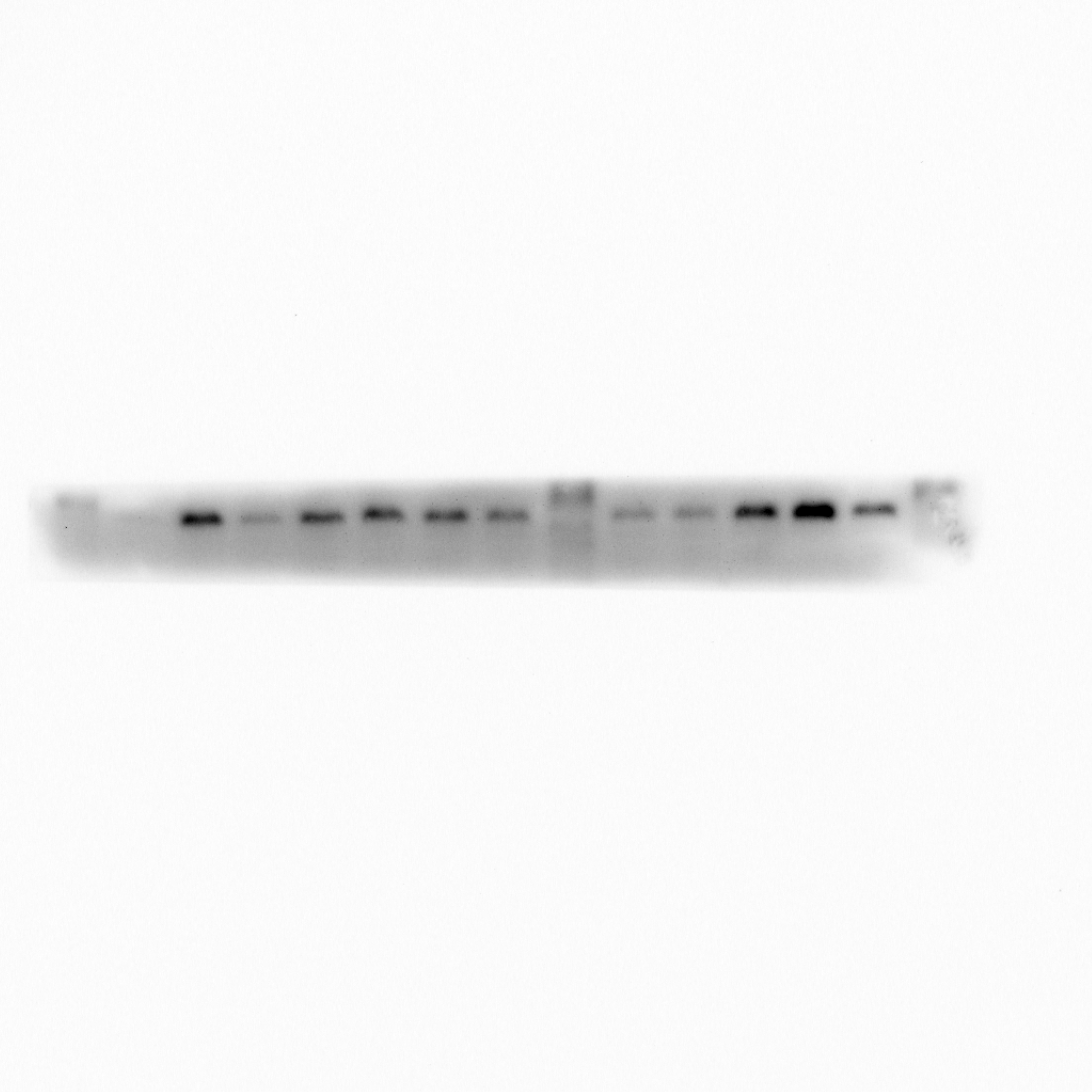

Supplement: Supplementary file 23 — Fig.6D-KLF4 [file 41420_2022_999_MOESM23_ESM.tif]

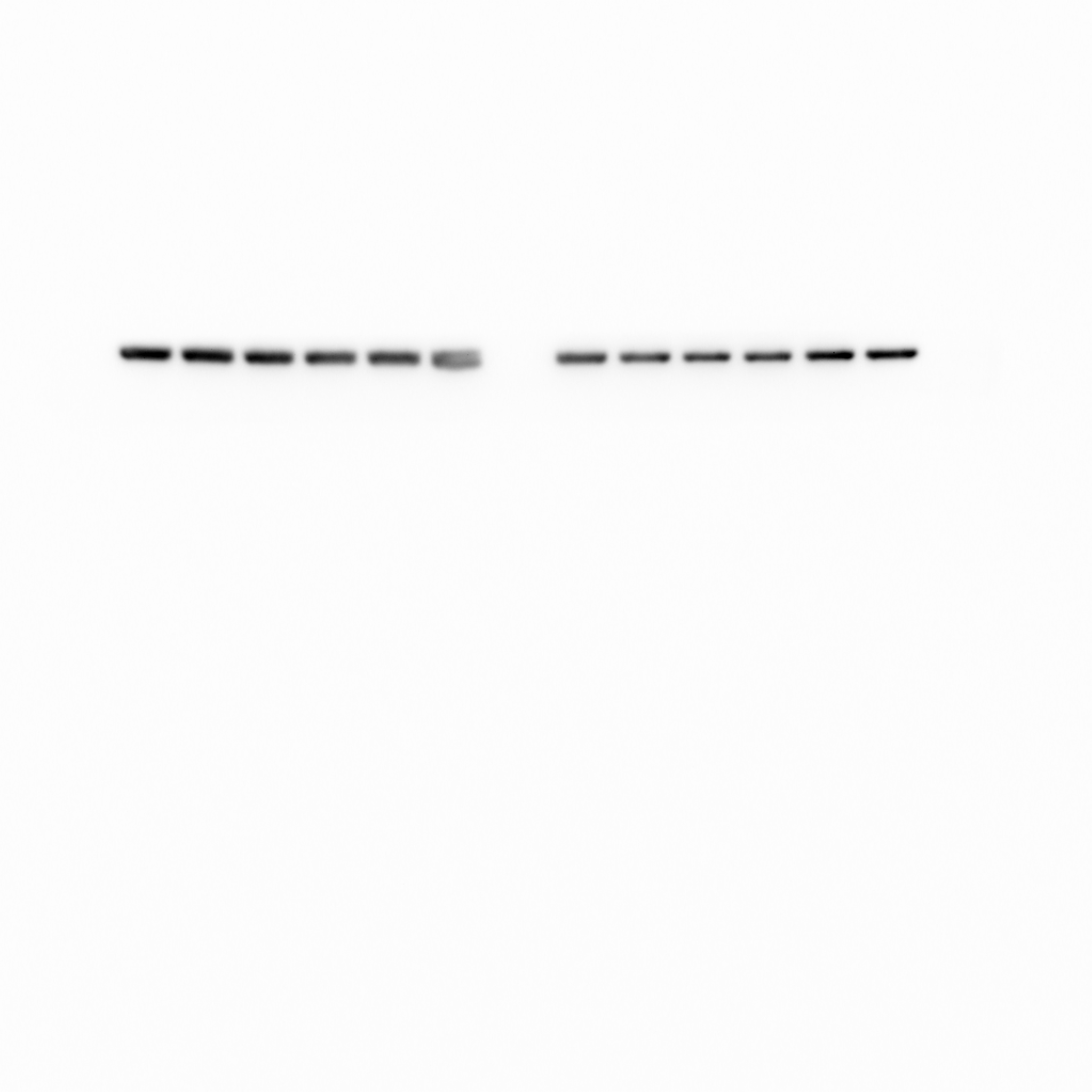

Supplement: Supplementary file 24 — Fig.6H-GAPDH for p110δ [file 41420_2022_999_MOESM24_ESM.tif]

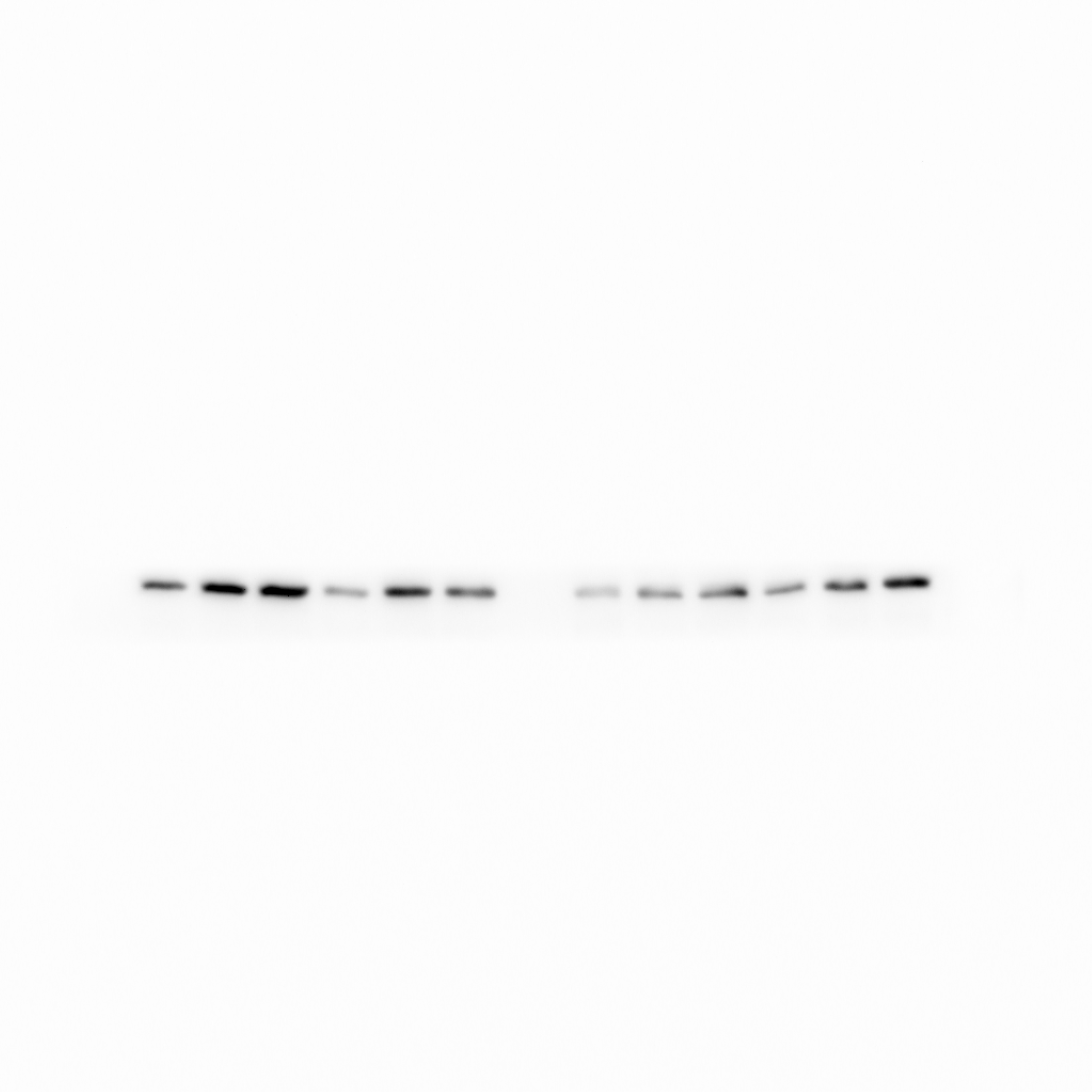

Supplement: Supplementary file 25 — Fig.6H-p110δ [file 41420_2022_999_MOESM25_ESM.tif]

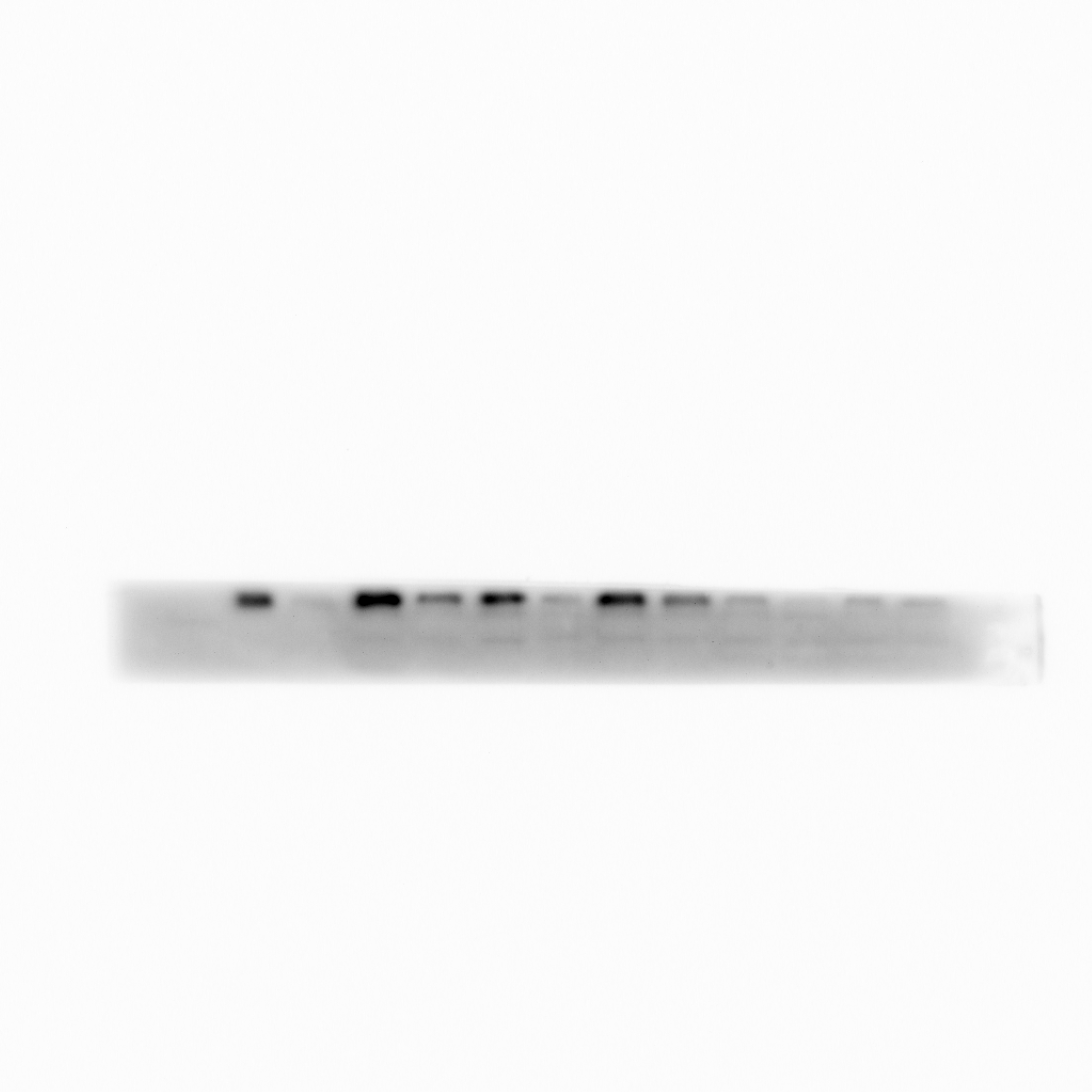

Supplement: Supplementary file 26 — Fig.6I-IκBα [file 41420_2022_999_MOESM26_ESM.tif]

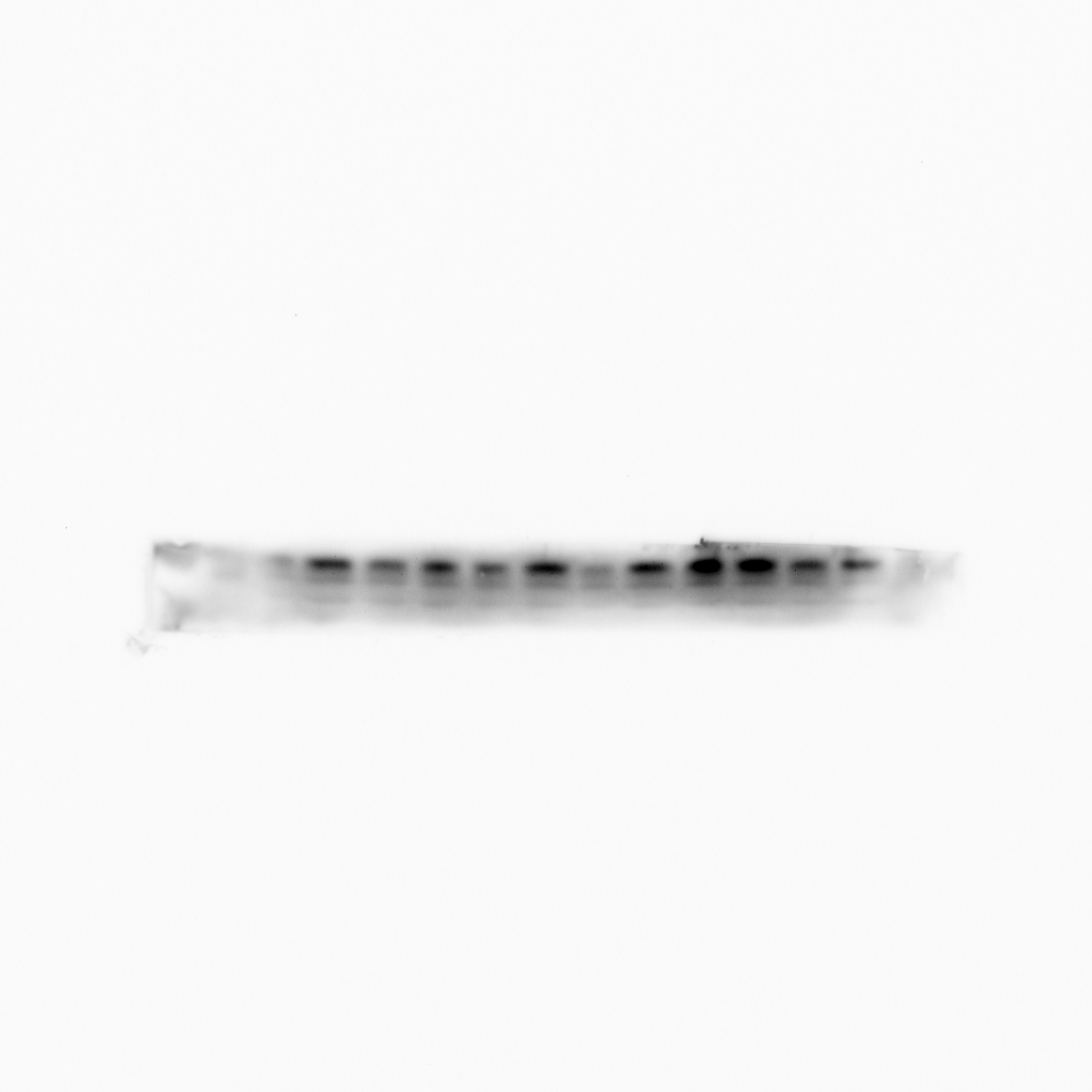

Supplement: Supplementary file 27 — Fig.6I-p-IκBα [file 41420_2022_999_MOESM27_ESM.tif]

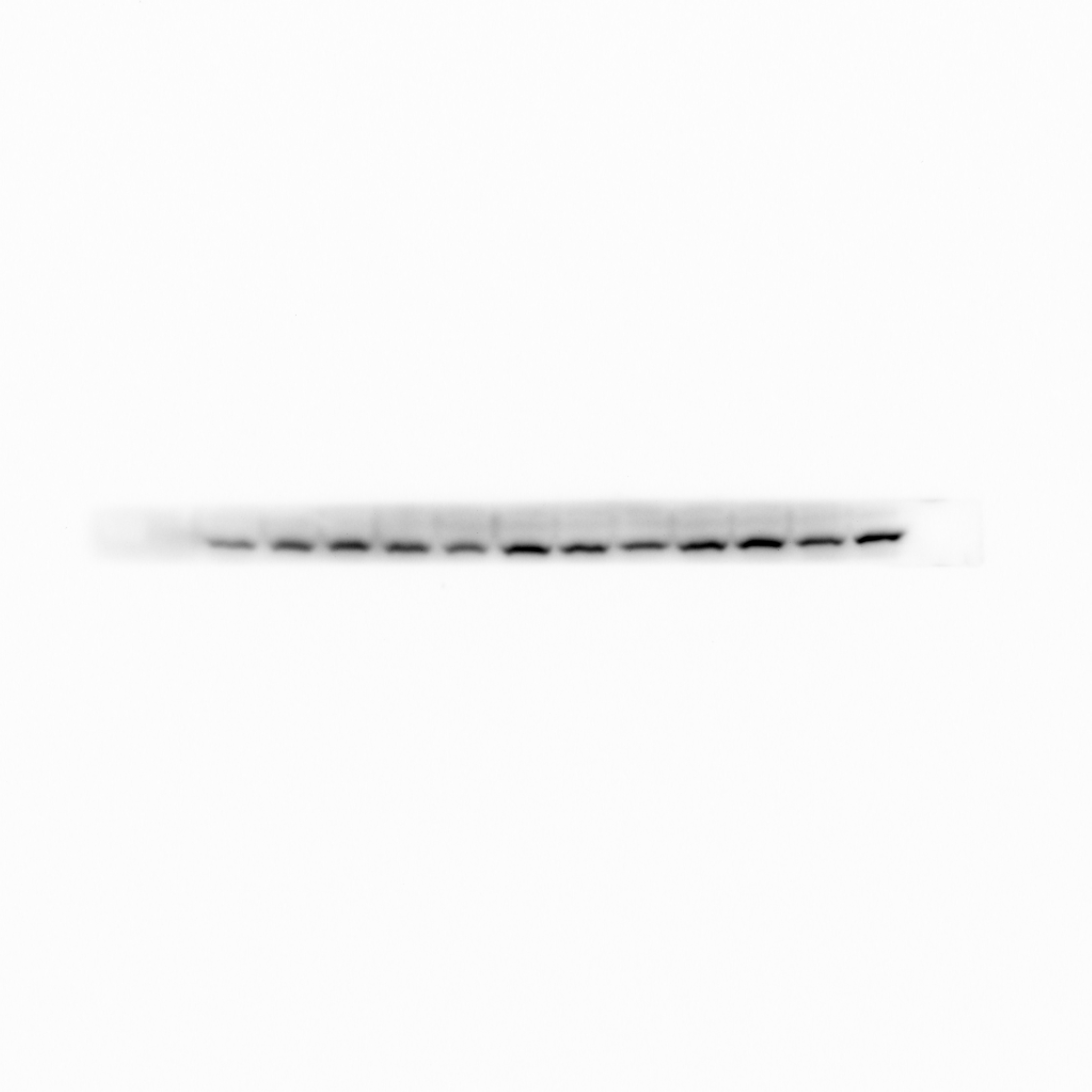

Supplement: Supplementary file 28 — Fig.6I-p-p65 [file 41420_2022_999_MOESM28_ESM.tif]

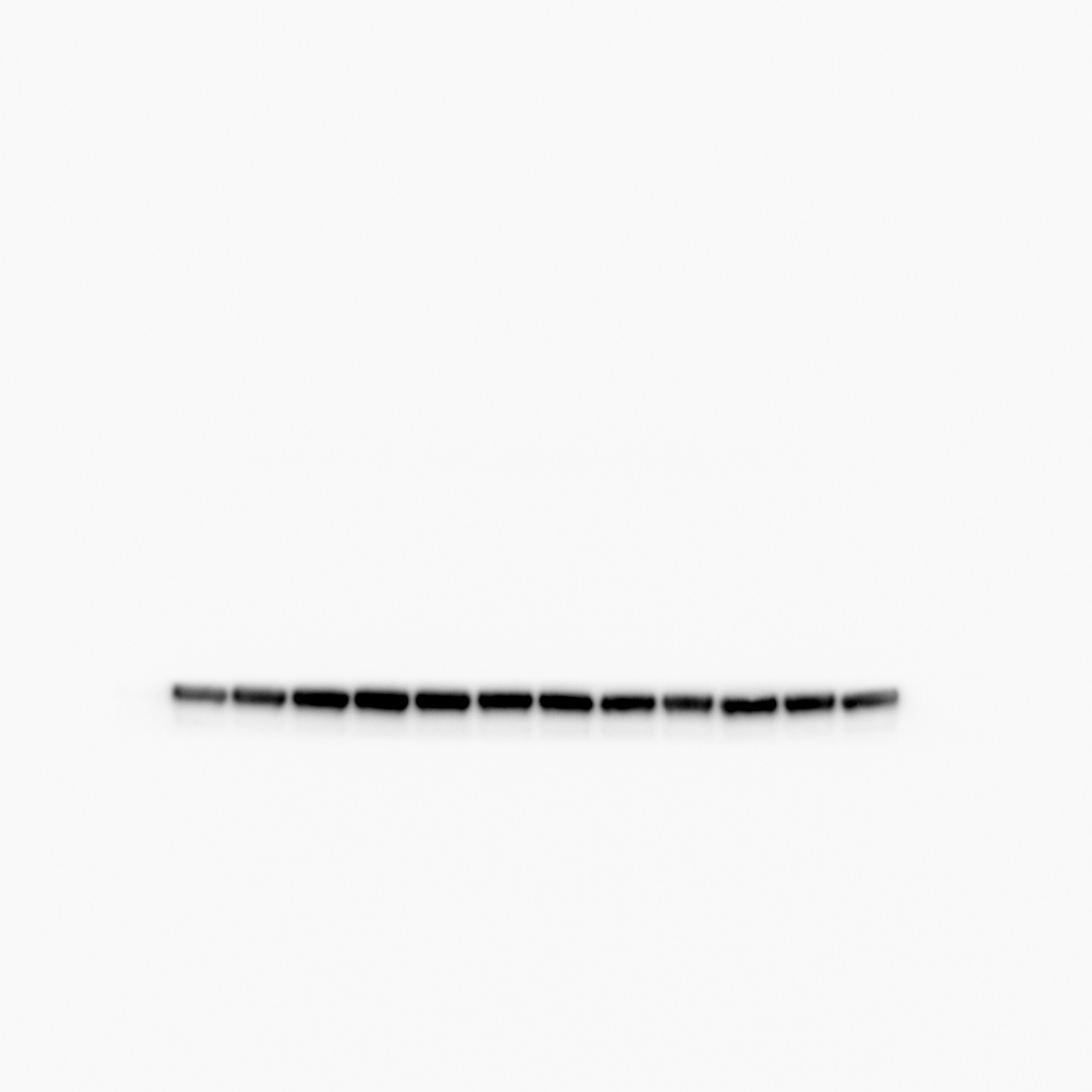

Supplement: Supplementary file 29 — Fig.6I-p65 [file 41420_2022_999_MOESM29_ESM.tif]

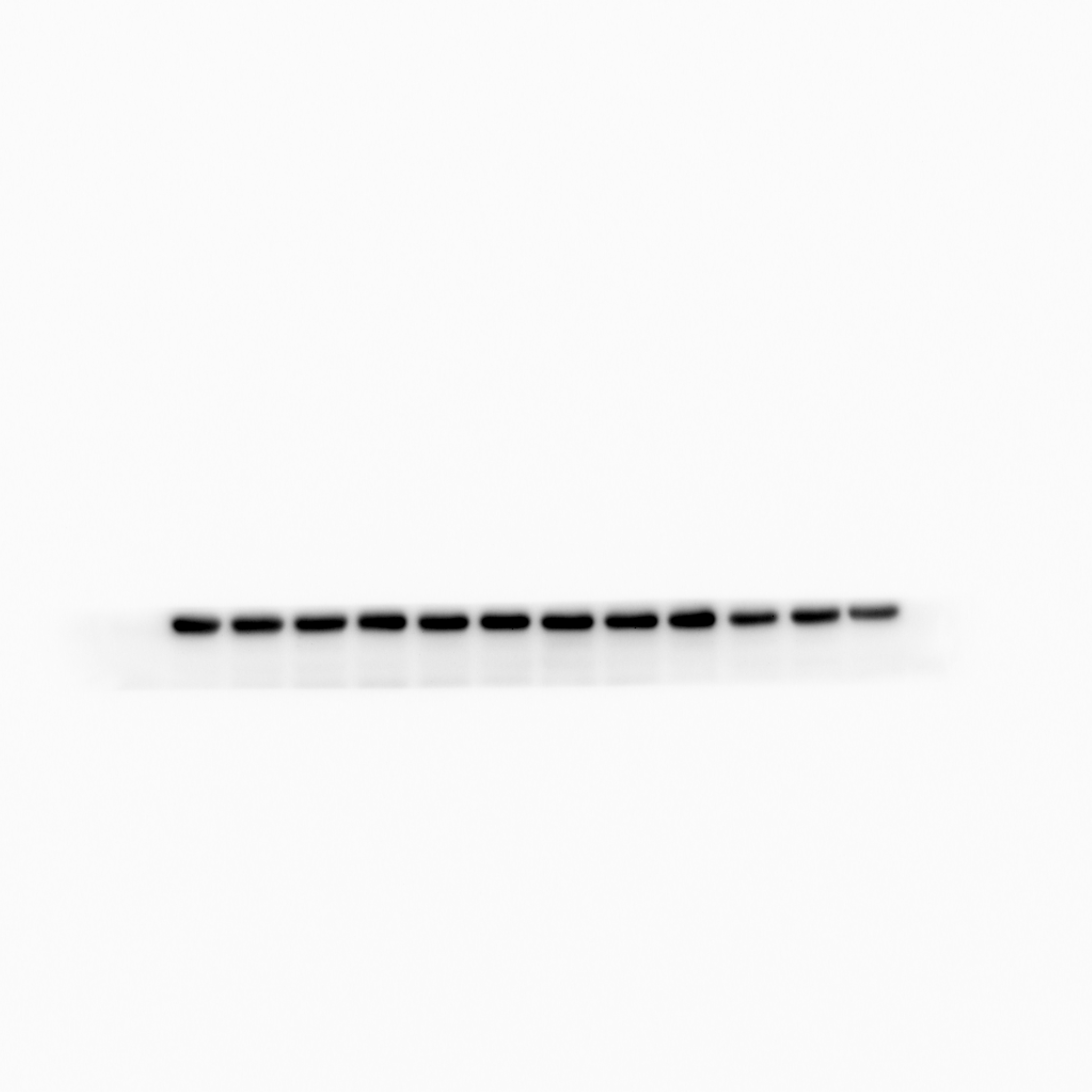

Supplement: Supplementary file 30 — Fig.6I-β Tubulin for NF-κB signaling pathway [file 41420_2022_999_MOESM30_ESM.tif]
