# Supplementary material for: MiR-92a/KLF4/p110δ regulates titanium particles-induced macrophages inflammation and osteolysis
Source: Cell Death Discov. 2022 Apr 13;8:197. doi: 10.1038/s41420-022-00999-2 (PMC9007998; doi:10.1038/s41420-022-00999-2)
Supplement: Supplementary file 31 — Supplemental material of figures and figures caption that are not shown in the manuscript [file 41420_2022_999_MOESM31_ESM.docx]

**Figure. S1**
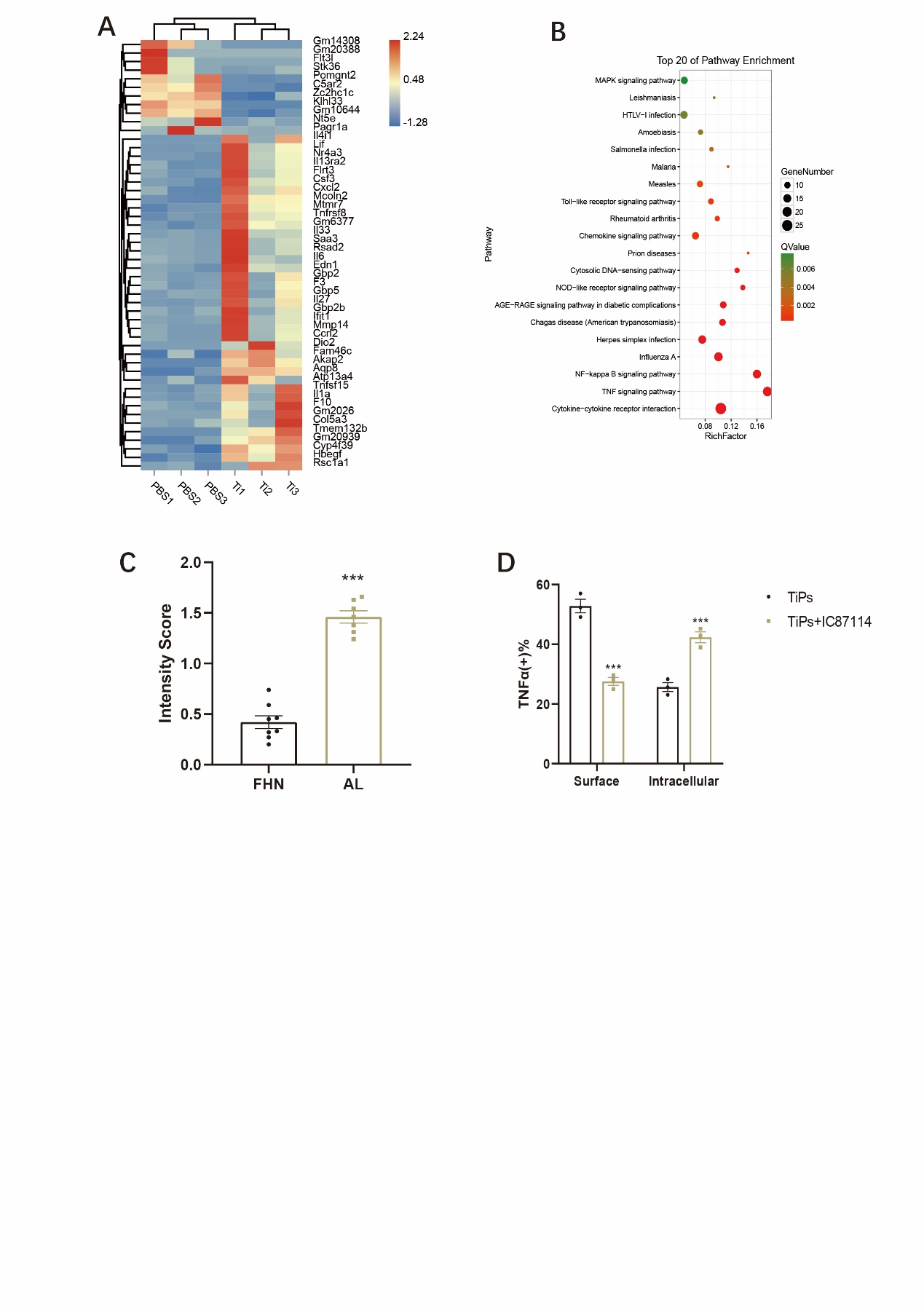


**Fig. S1 RNA-seq analysis and quantitative analysis of IHC staining result of p110δ in synovial membranes. (A)** Heat map presenting the top 50 differentially expressed genes (DEGs). **(B)**The top 20 pathways enrichment was portrayed after RNA-seq analysis of 4h TiPs activated RAW264.7. **(C)** Average intensity score (IS) of p110δ IHC staining was calculated by Bresalier’s analysis. **(D)** Quantitative analysis of FCM assay to detect surface and intracellular TNFα after 2h TiPs stimulation with or without 10μM IC87114. For surface TNFα detection, permeabilization was not applied and 10μM TAPI-1 (TACE inhibitor) was employed to block the cleavage of TNFα. The surface TNFα detection could reflect the secretion of TNFα. For intracellular detection, permeabilization was conducted without TAPI-1 treatment. And the intracellular detection could reflect the cellular residual TNFα. All data were concluded from at least three independent assays. Statistic data were displayed as mean ± SEM and were conducted unpaired t test analysis to determine significant difference. * *p*< 0.05, ** *p*< 0.01, *** *p*< 0.001 compared with the negative group.

**Figure. S2**


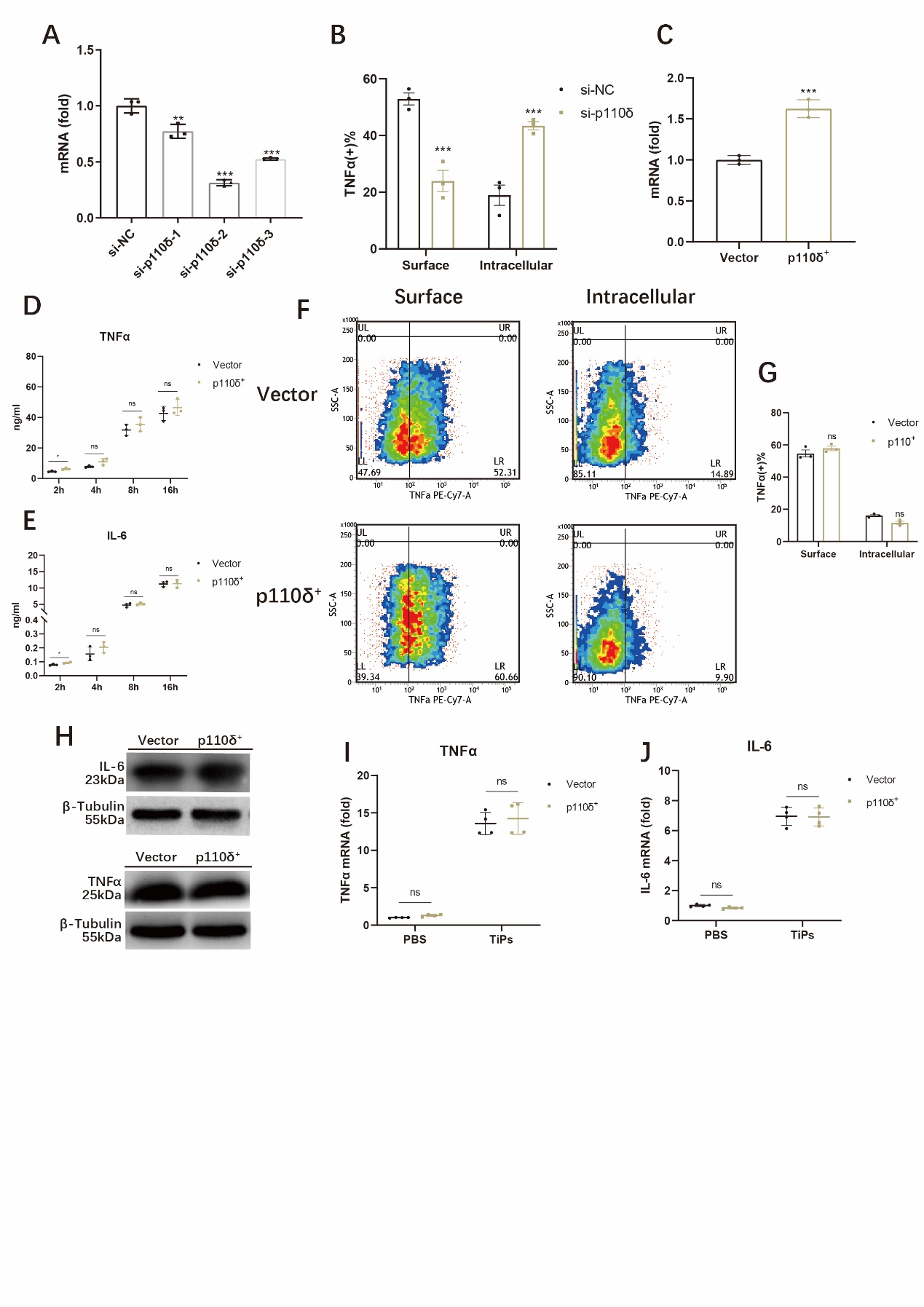


**Fig. S2 TiPs-induced inflammation in p110δ-altered macrophages.** **(A)** mRNA expression of p110δ from macrophages upon three different siRNAs transfection. **(B)** Quantitative analysis of FCM assay to analyse TNFα production in si-NC or si-p110δ transfected macrophages upon TiPs stimulation **(C)** mRNA expression of p110δ in p110δ-overexpressed macrophages. **(D, E)** TiPs-induced secretions of TNFα **(D)** and IL-6 **(E)** were detected by ELISA assay upon overexpression of p110δ. **(F)** The surface TNFα and intracellular TNFα were analyzed respectively by FCM assay after 2h TiPs stimulation. For surface TNFα detection, permeabilization was not applied and 10μM TAPI-1 (TACE inhibitor) was employed to block the cleavage of TNFα. The surface TNFα detection could reflect the secretion of TNFα. For intracellular detection, permeabilization was conducted without TAPI-1 treatment. And the intracellular detection could reflect the cellular residual TNFα **(G)** Quantitative analysis of FCM assay to detect TNFα production in Vector or p110δ^+^ transfected macrophages upon TiPs stimulation. **(H)** Co-treated with BFA, 8h TiPs activated macrophages were performed WB assay to exam protein levels of TNFα and IL-6. **(I, J)** mRNA expressions of TNFα **(I)** and IL-6 **(J)** were examined by qPCR assay in p110δ-overexpressed RAW264.7 under 8h TiPs stimulation. All data were concluded from at least three independent assays. Statistic data were displayed as mean ± SEM and were conducted unpaired t test analysis or one-way ANOVA analysis or two-way ANOVA analysis to determine significant difference. * *p*< 0.05, ** *p*< 0.01, *** *p*< 0.001 compared with the negative group.


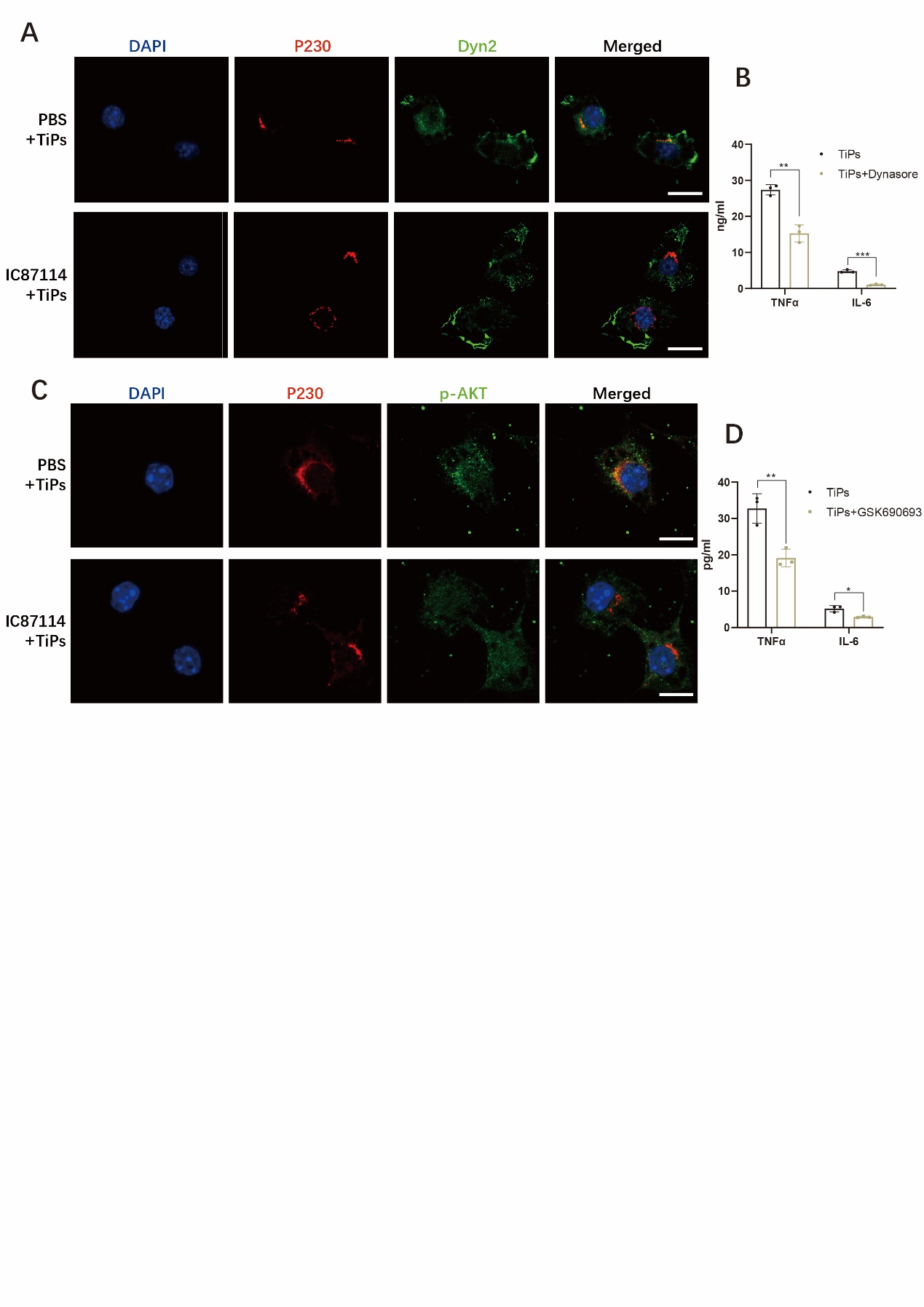
**Figure. S3**

**Fig. S3 Dyn2 and AKT might act as the downstream factors of p110δ. (A)** After stimulated with 2h TiPs with or without IC87114, RAW264.7 were imaged by confocal microscopy to detect the distribution of Dyn2 and p230. The white bar, 10μm. **(B)** The impact of Dynasore, inhibitor of Dyn2, on 8h TiPs-induced TNFα and IL-6 secretions. **(C)** After stimulated with 2h TiPs with or without IC87114, RAW264.7 were imaged by confocal microscopy to detect the distribution of p-AKT and p230. The white bar, 10μm. **(D)** The impact of GSK690693, pan-AKT inhibitor, on 8h TiPs-induced TNFα and IL-6 secretions. All data were concluded from at least three independent assays. Statistic data were displayed as mean ± SEM and were conducted unpaired t test analysis to determine significant difference. * *p*< 0.05, ** *p*< 0.01, *** *p*< 0.001 compared with the negative group.


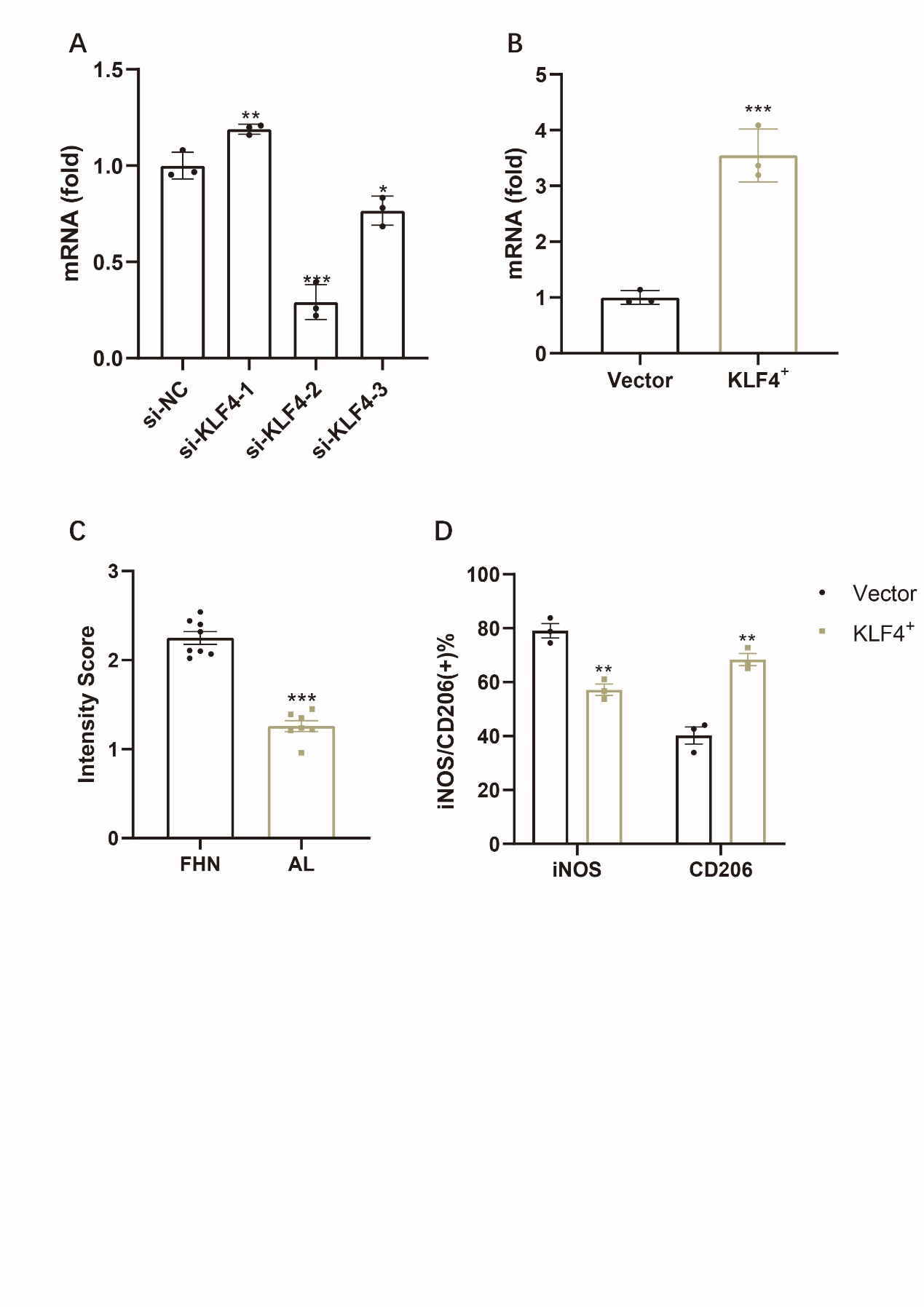
**Figure. S4**

**Fig. S4 TiPs-induced inflammation in KLF4-altered macrophages and average intensity score (IS) of KLF4 IHC staining result in synovial membranes. (A)** mRNA expression of KLF4 from macrophages upon three different siRNAs transfection. **(B)** mRNA expression of KLF4 from macrophages transfected with KLF4-overexpressed plasmids. **(C)** Synovial membranes from femoral head necrosis (FHN) patients achieved a higher average intensity score (IS) of KLF4 staining than those from prosthetic aseptic loosening (AL) patients. **(D)** Quantitative analysis of FCM analysis to detect iNOS or CD206 production in Vector or KLF4^+^ transfected macrophages upon 24h TiPs stimulation. All data were concluded from at least three independent assays. Statistic data were displayed as mean ± SEM and were conducted unpaired t test analysis or one-way ANOVA analysis to determine significant difference. * *p*< 0.05, ** *p*< 0.01, *** *p*< 0.001 compared with the negative group.


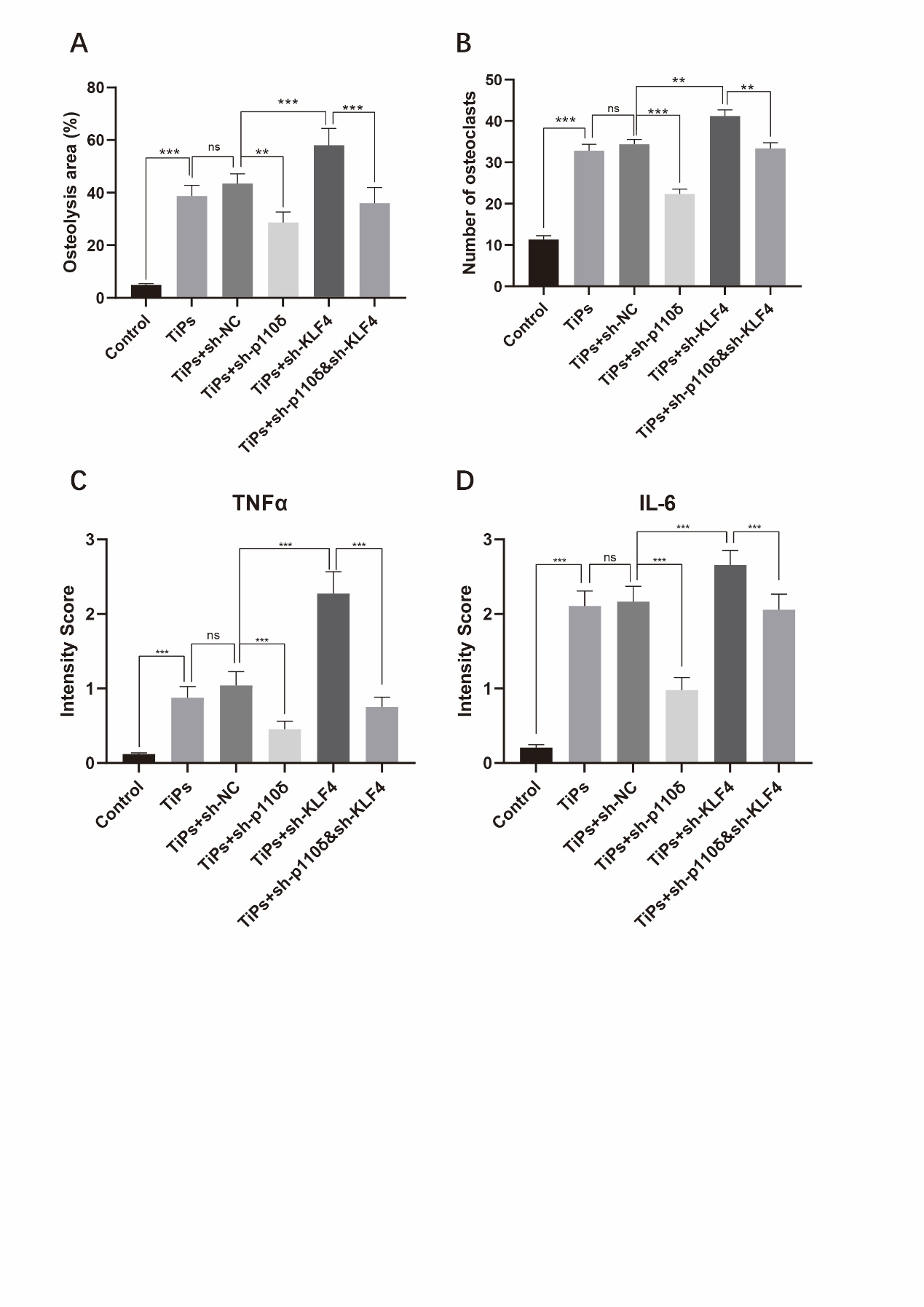
**Figure. S5**

**Fig. S5 Quantitative analysis of osteolysis area and number of osteoclasts in TiPs-induced mice cranial osteolysis.** **(A)** Quantitative analysis of osteolysis area. **(B)** Quantitative analysis of number of osteoclasts. **(C)** Average intensity score (IS) of TNFα IHC staining of mice calvarias was calculated by Bresalier’s analysis. **(D)** Average intensity score (IS) of IL-6 IHC staining of mice calvarias was calculated by Bresalier’s analysis. Statistic data were displayed as mean ± SEM and were conducted one-way ANOVA analysis to determine significant difference. * *p*< 0.05, ** *p*< 0.01, *** *p*< 0.001 compared with the negative group
